# Supplementary figures and images for: Characterizing nitrogen cycling microorganisms and genes in sediments of the Three Gorges Reservoir
Source: PLoS One. 2025 Jun 10;20(6):e0324051. doi: 10.1371/journal.pone.0324051 (PMC12151472; doi:10.1371/journal.pone.0324051)

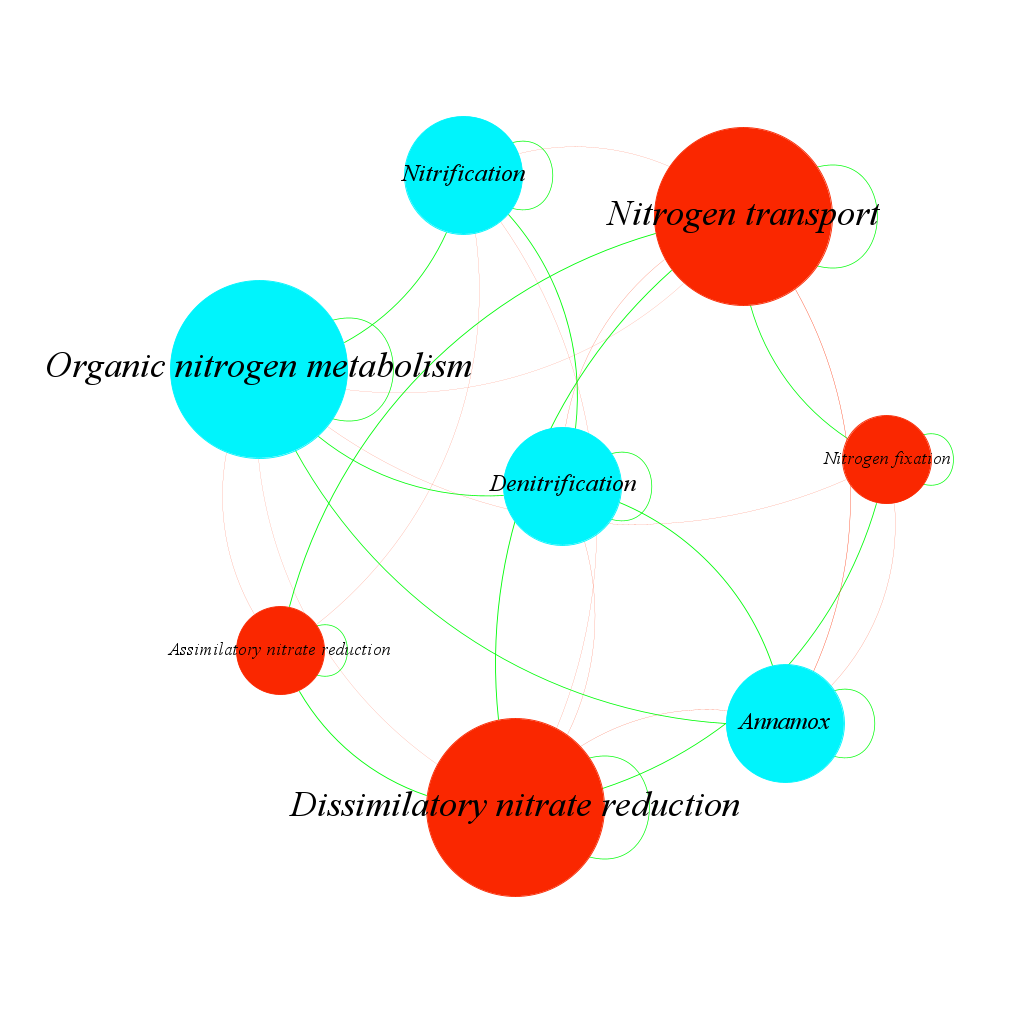

Supplement: S1 File — (ZIP) [file pone.0324051.s001.zip › Dataset/Network Analysis Diagram/Function/Downstream/network_function_downstream.png]

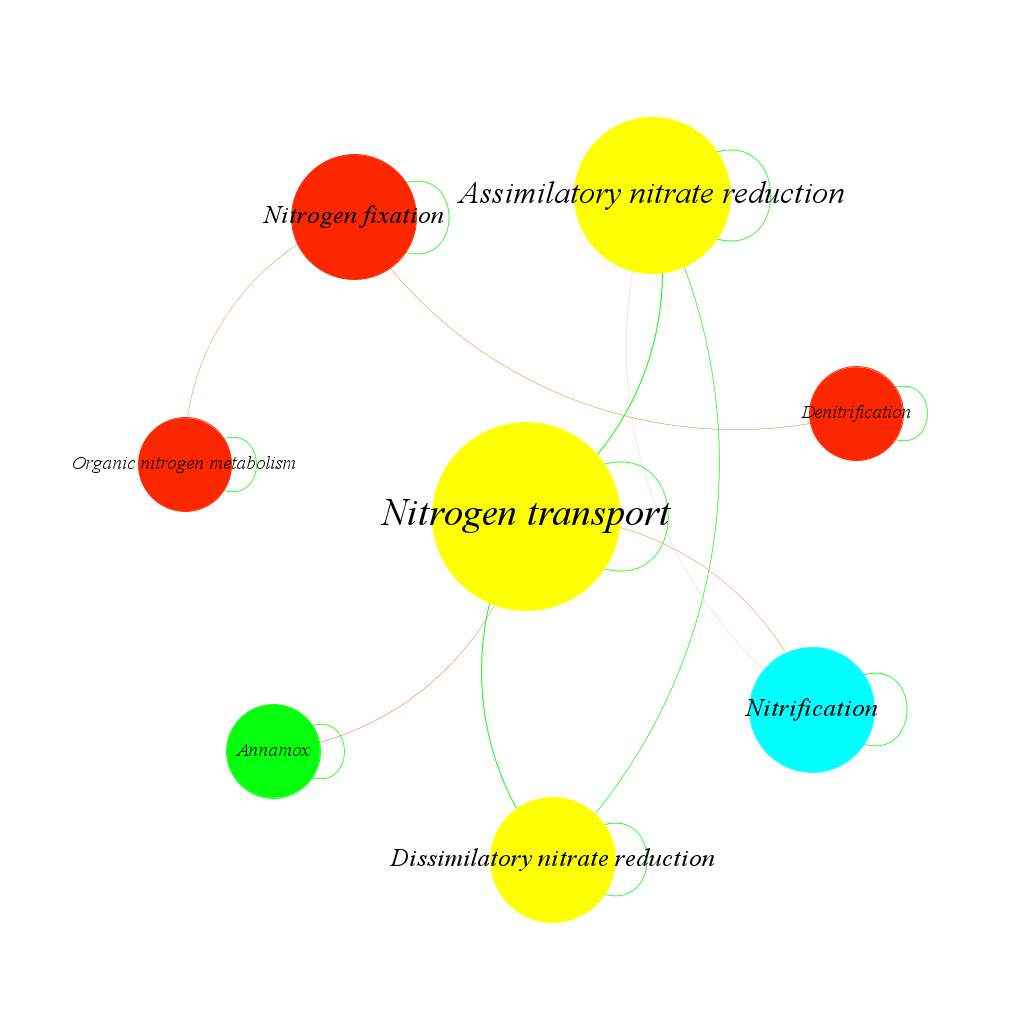

Supplement: S1 File — (ZIP) [file pone.0324051.s001.zip › Dataset/Network Analysis Diagram/Function/Midstream/network_function_midstream.png]

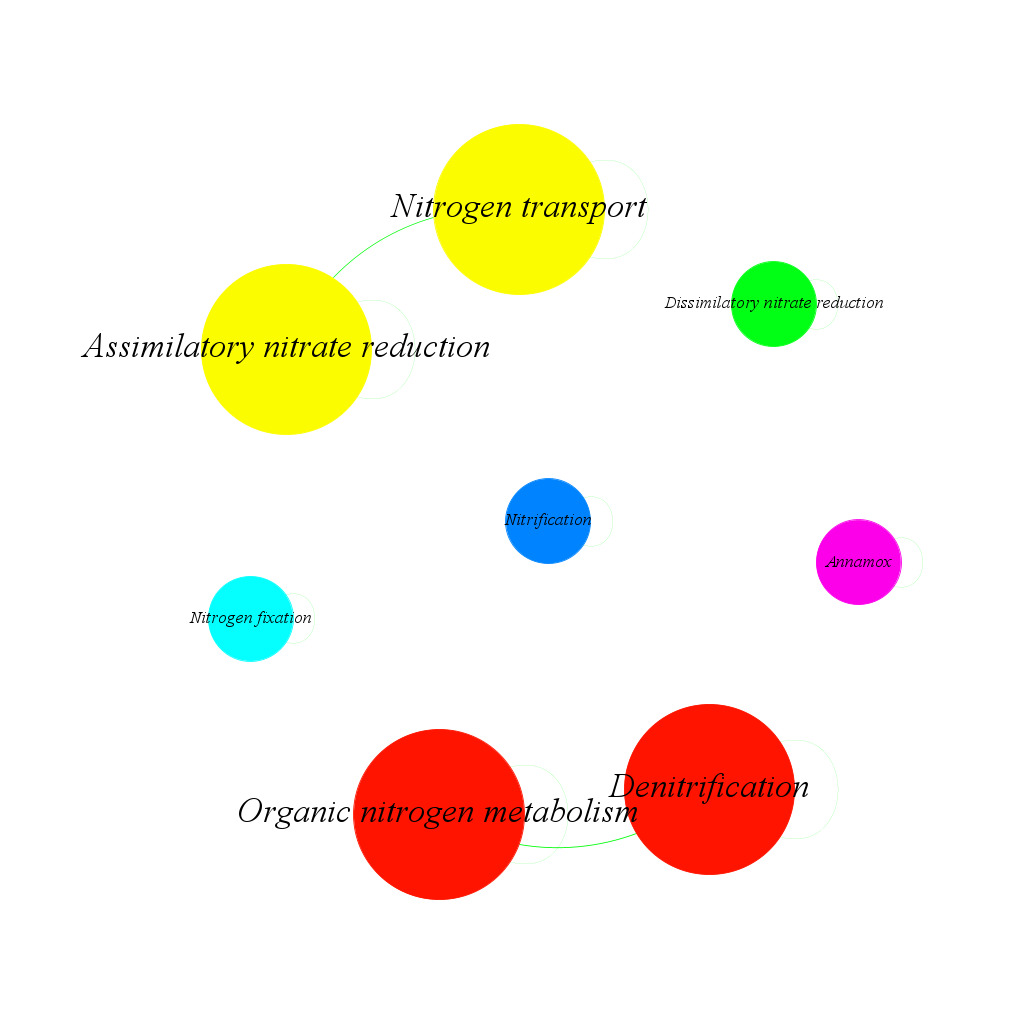

Supplement: S1 File — (ZIP) [file pone.0324051.s001.zip › Dataset/Network Analysis Diagram/Function/Upstream/network_function_upstream.png]

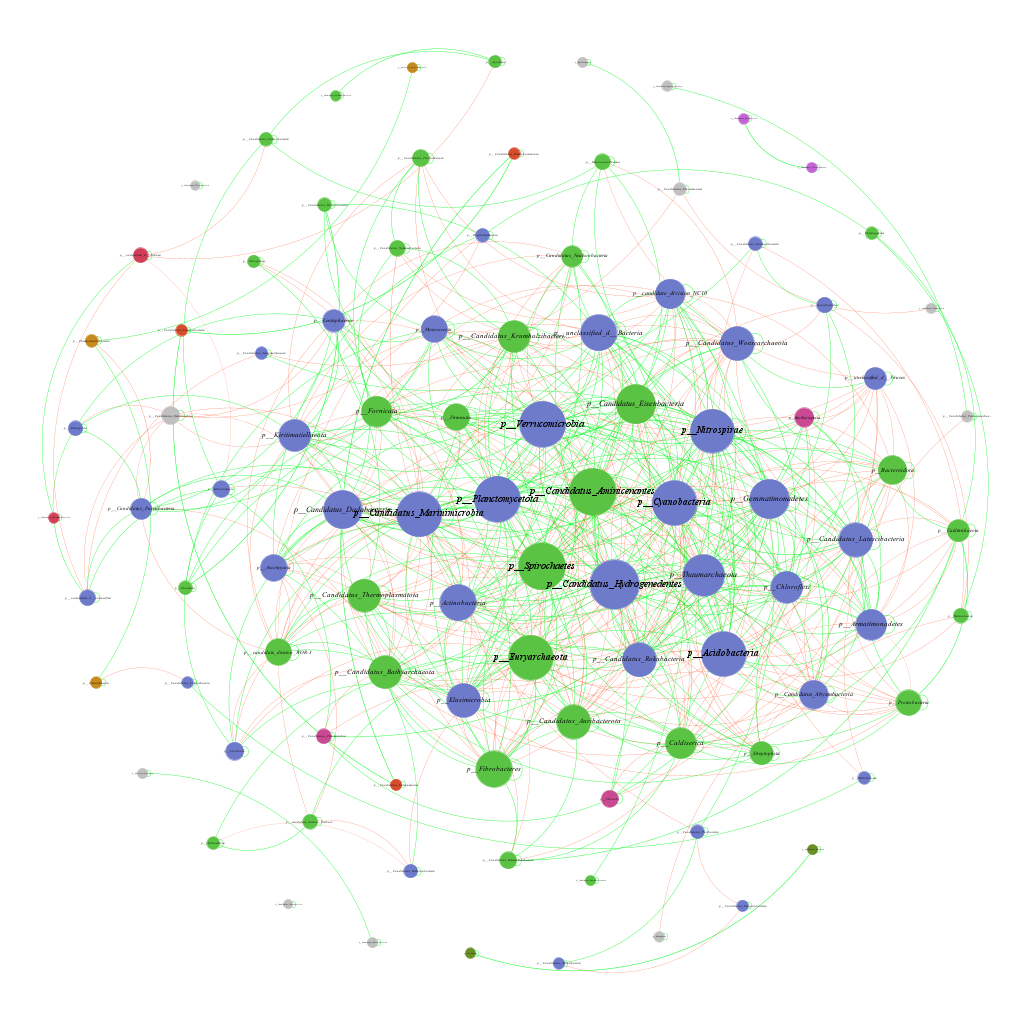

Supplement: S1 File — (ZIP) [file pone.0324051.s001.zip › Dataset/Network Analysis Diagram/Species/Downstream/network_species_downstream.png]

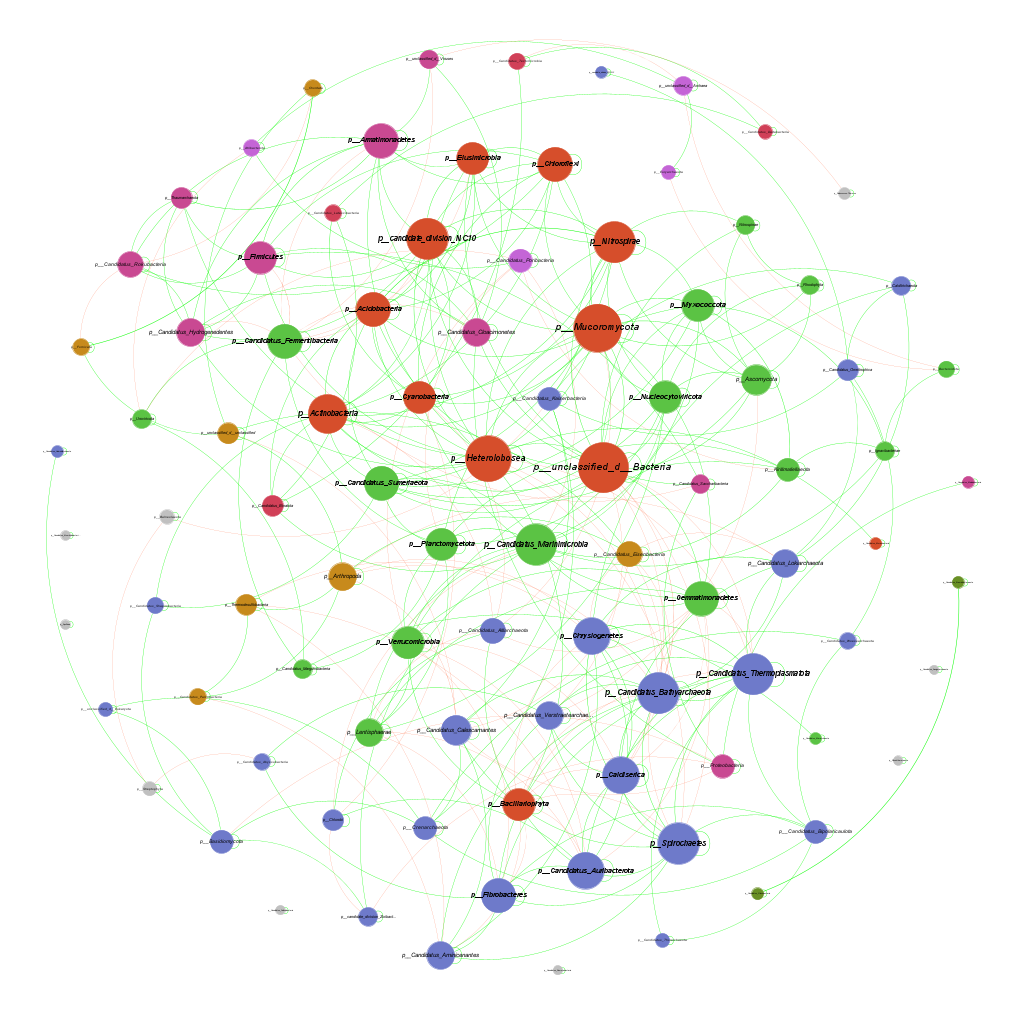

Supplement: S1 File — (ZIP) [file pone.0324051.s001.zip › Dataset/Network Analysis Diagram/Species/Midstream/network_species_midstream.png]

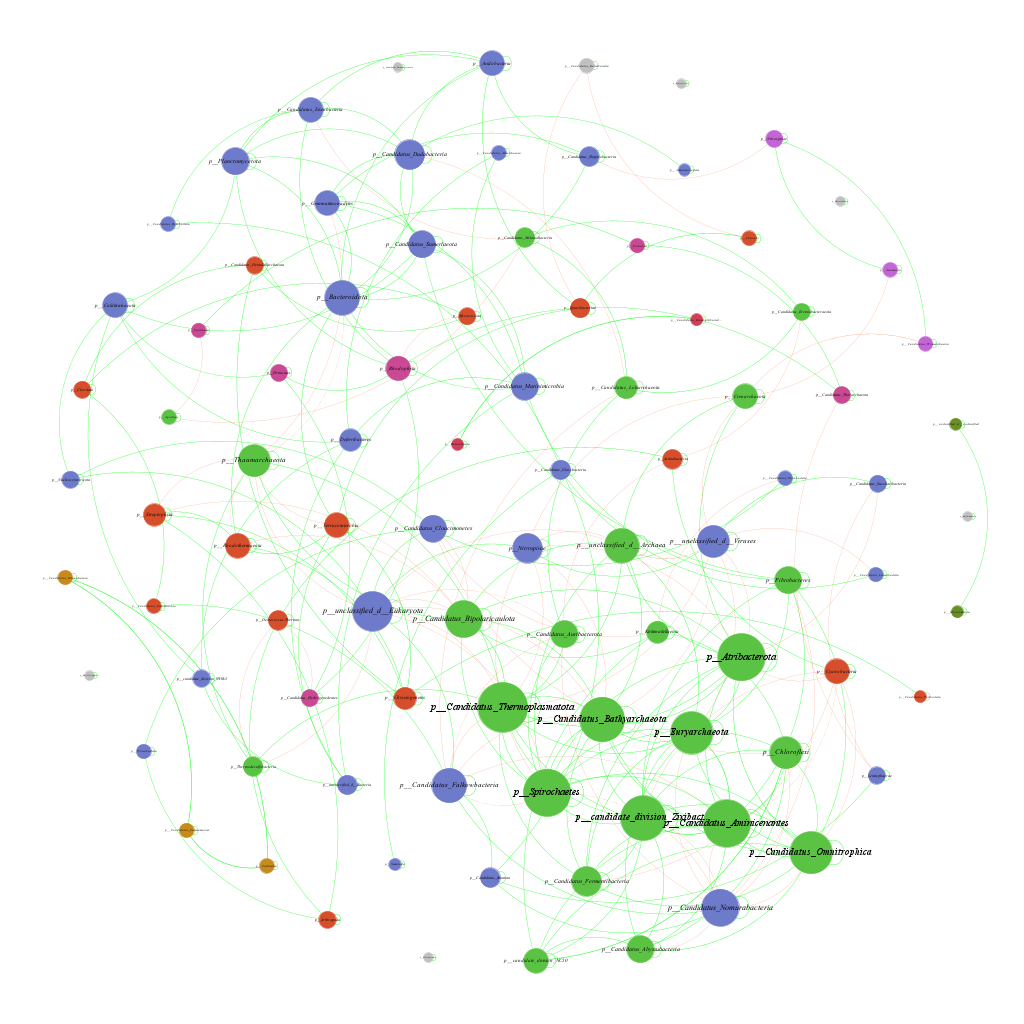

Supplement: S1 File — (ZIP) [file pone.0324051.s001.zip › Dataset/Network Analysis Diagram/Species/Upstream/network_species_upstream.png]
