# Supplementary material for: Characterizing nitrogen cycling microorganisms and genes in sediments of the Three Gorges Reservoir
Source: PLoS One. 2025 Jun 10;20(6):e0324051. doi: 10.1371/journal.pone.0324051 (PMC12151472; doi:10.1371/journal.pone.0324051)
Supplement: Attachment — Submitted filename: PONE-D-24-37897 (1).pdf [file pone.0324051.s002.pdf]

PLOS ONE

Characterizing Nitrogen-Cycling Microorganisms and Genes in Sediments of the Three Gorges Reservoir

--Manuscript Draft--

**Manuscript Number:**	PONE-D-24-37897
**Article Type:**	Research Article
**Full Title:**	Characterizing Nitrogen-Cycling Microorganisms and Genes in Sediments of the Three Gorges Reservoir
**Short Title:**	No short title
**Corresponding Author:**	Wenjun Yang<br>Changjiang River Scientific Research Institute<br>Wuhan, Hubei CHINA
**Keywords:**	Three Gorges Reservoir; Sediment; Nitrogen Cycle; Metagenomics
**Abstract:**	Microorganisms play a central role in driving the biogeochemical cycles in lakes (reservoirs). This study aims to refine the microbial-driven nitrogen cycle processes in the sediments of the Three Gorges Reservoir and assess the overall state of nitrogen cycling within these sediments. The study focuses on the Three Gorges Reservoir as the research area, systematically investigates the nitrogen cycle microorganisms and corresponding functional gene abundance characteristics attached to sediments from upstream, midstream, and downstream areas within the region, and explores key factors that may influence the composition of nitrogen cycle microbial communities. The primary findings of this paper include: 46 types of nitrogen conversion functional genes were annotated in the Three Gorges Reservoir sediment samples, covering seven major nitrogen cycle pathways: nitrogen fixation, nitrification, denitrification, nitrogen transport, organic nitrogen metabolism, assimilatory nitrate reduction, and dissimilatory nitrate reduction. The abundance of anaerobic ammonia oxidation functional genes is extremely low (almost zero), indicating that there may be no anaerobic ammonia oxidation potential on the particulate matter; there are differences in the abundance of nitrogen cycle functional genes and the composition of nitrogen cycle microbial communities among sediments with different particle sizes from upstream, midstream, and downstream areas, suggesting that sediment particle size may affect the composition of nitrogen cycle microbial communities attached to it; as sediment particle size decreases, the α and β diversity of nitrogen cycle microorganisms significantly increases, indicating that sediment particle size may have a significant impact on the diversity of nitrogen cycle microorganisms.
**Order of Authors:**	Chang Han
	Zhiyuan Chen
	Yihui Xiao
	Ting Yang
	Haoyang Shi
	Huiqun Cao
	Wenjun Yang
**Additional Information:**	
**Question**	**Response**
**Financial Disclosure**<br><br>Enter a financial disclosure statement that describes the sources of funding for the work included in this submission. Review the submission guidelines for detailed	Yes

Powered by Editorial Manager® and ProduXion Manager® from Aries Systems Corporation

requirements. View published research articles from *PLOS ONE* for specific examples.<br><br>This statement is required for submission and **will appear in the published article** if the submission is accepted. Please make sure it is accurate.<br><br>**Funded studies**<br>Enter a statement with the following details:<br>• Initials of the authors who received each award<br>• Grant numbers awarded to each author<br>• The full name of each funder<br>• URL of each funder website<br>• Did the sponsors or funders play any role in the study design, data collection and analysis, decision to publish, or preparation of the manuscript?<br><br>Did you receive funding for this work?	
Please add funding details.<br>as follow-up to "**Financial Disclosure**<br><br>Enter a financial disclosure statement that describes the sources of funding for the work included in this submission. Review the submission guidelines for detailed requirements. View published research articles from *PLOS ONE* for specific examples.<br><br>This statement is required for submission and **will appear in the published article** if the submission is accepted. Please make sure it is accurate.	The authors gratefully acknowledge the financial support provided by the Key Project of the National Natural Science Foundation of China (52130903).

**Funded studies**<br>Enter a statement with the following details:<br>• Initials of the authors who received each award<br>• Grant numbers awarded to each author<br>• The full name of each funder<br>• URL of each funder website<br>• Did the sponsors or funders play any role in the study design, data collection and analysis, decision to publish, or preparation of the manuscript?<br><br>Did you receive funding for this work?"	
Please select the country of your main research funder (please select carefully as in some cases this is used in fee calculation).<br>as follow-up to "**Financial Disclosure**<br><br>Enter a financial disclosure statement that describes the sources of funding for the work included in this submission. Review the submission guidelines for detailed requirements. View published research articles from *PLOS ONE* for specific examples.<br><br>This statement is required for submission and **will appear in the published article** if the submission is accepted. Please make sure it is accurate.<br><br>**Funded studies**<br>Enter a statement with the following details:<br>• Initials of the authors who received each award<br>• Grant numbers awarded to each author<br>• The full name of each funder<br>• URL of each funder website<br>• Did the sponsors or funders play any role in the study design, data collection and analysis, decision to publish, or preparation of the manuscript?<br><br>Did you receive funding for this work?"	CHINA - CN
**Competing Interests**	The articles I submit are completely original, without any act of plagiarism or improper

Use the instructions below to enter a competing interest statement for this submission. On behalf of all authors, disclose any competing interests that could be perceived to bias this work—acknowledging all financial support and any other relevant financial or non-financial competing interests.<br><br>This statement is required for submission and **will appear in the published article** if the submission is accepted. Please make sure it is accurate and that any funding sources listed in your Funding Information later in the submission form are also declared in your Financial Disclosure statement.<br><br>View published research articles from *PLOS ONE* for specific examples.<br><br>**NO authors have competing interests**<br><br>Enter: *The authors have declared that no competing interests exist.*<br><br>**Authors with competing interests**<br><br>Enter competing interest details beginning with this statement:<br><br>*I have read the journal's policy and the authors of this manuscript have the following competing interests: [insert competing interests here]*<br><br>* typeset	quotation. The articles submitted in this article are completed by me in the process of study, research and work, and there is no conflict of interest. I do not receive any relevant honors and compensation for this. I have clearly understood that the copyright of the submitted articles belongs to this journal, and this journal has the right to copy, disseminate and digitally store the relevant articles. I have clearly understood that the publication of my articles in this journal indicates the approval of this article, but the articles published in this journal are for academic communication only and shall not be used for commercial purposes. I guarantee that the articles submitted have not been published in academic institutions at home and abroad, and will not be re-submitted or reproduced in any other academic institutions during or after the submission. I guarantee that if the submitted article has been submitted or published in other journals, I should provide the relevant publication certificate and clearly indicate the difference between the cited literature and this article, so as to prevent the relevant article from bringing relevant conflicts of interest when it is published in this journal. If there is any necessary copyright statement in the submission of this article, I guarantee that it will be clearly marked in the article submission and ensure that there is a correct copyright statement to avoid copyright disputes caused by the article after publication. If the relevant conflict of interest is caused by my article after publication, I agree to be responsible for resolving it and will not hold this journal or any related personnel responsible. I promise that any terms and content of this statement are true, legal and valid. If it is not true, I will bear all consequences arising therefrom.
**Ethics Statement**<br><br>Enter an ethics statement for this submission. This statement is required if the study involved:<br><br>• Human participants<br>• Human specimens or tissue<br>• Vertebrate animals or cephalopods<br>• Vertebrate embryos or tissues	N/A

• Field research<br><br>Write "N/A" if the submission does not require an ethics statement.<br><br>General guidance is provided below. Consult the submission guidelines for detailed instructions. **Make sure that all information entered here is included in the Methods section of the manuscript.**	

**Format for specific study types**<br><br>**Human Subject Research (involving human participants and/or tissue)**<br>• Give the name of the institutional review board or ethics committee that approved the study<br>• Include the approval number and/or a statement indicating approval of this research<br>• Indicate the form of consent obtained (written/oral) or the reason that consent was not obtained (e.g. the data were analyzed anonymously)<br><br>**Animal Research (involving vertebrate animals, embryos or tissues)**<br>• Provide the name of the Institutional Animal Care and Use Committee (IACUC) or other relevant ethics board that reviewed the study protocol, and indicate whether they approved this research or granted a formal waiver of ethical approval<br>• Include an approval number if one was obtained<br>• If the study involved *non-human primates*, add *additional details* about animal welfare and steps taken to ameliorate suffering<br>• If anesthesia, euthanasia, or any kind of animal sacrifice is part of the study, include briefly which substances and/or methods were applied<br><br>**Field Research**<br><br>Include the following details if this study involves the collection of plant, animal, or other materials from a natural setting:<br>• Field permit number<br>• Name of the institution or relevant body that granted permission	
**Data Availability**<br><br>Authors are required to make all data underlying the findings described fully available, without restriction, and from the time of publication. PLOS allows rare exceptions to address legal and ethical concerns. See the PLOS Data Policy and FAQ for detailed information.	Yes - all data are fully available without restriction

A Data Availability Statement describing where the data can be found is required at submission. Your answers to this question constitute the Data Availability Statement and **will be published in the article**, if accepted.<br><br>**Important:** Stating 'data available on request from the author' is not sufficient. If your data are only available upon request, select 'No' for the first question and explain your exceptional situation in the text box.<br><br>Do the authors confirm that all data underlying the findings described in their manuscript are fully available without restriction?	
**Describe where the data may be found in full sentences. If you are copying our sample text, replace any instances of XXX with the appropriate details.**<br><br>• If the data are **held or will be held in a public repository**, include URLs, accession numbers or DOIs. If this information will only be available after acceptance, indicate this by ticking the box below. For example: *All XXX files are available from the XXX database (accession number(s) XXX, XXX.*).<br>• If the data are all contained **within the manuscript and/or Supporting Information files**, enter the following: *All relevant data are within the manuscript and its Supporting Information files.*<br>• If neither of these applies but you are able to provide **details of access elsewhere**, with or without limitations, please do so. For example:<br><br>*Data cannot be shared publicly because of [XXX]. Data are available from the XXX Institutional Data Access / Ethics Committee (contact via XXX) for researchers who meet the criteria for access to confidential data.*<br><br>*The data underlying the results presented in the study are available from (include the name of the third party*	All relevant data are within the manuscript and its Supporting Information files.

and contact information or URL).<br>• This text is appropriate if the data are owned by a third party and authors do not have permission to share the data.<br><br>* typeset	
Additional data availability information:	Tick here if the URLs/accession numbers/DOIs will be available only after acceptance of the manuscript for publication so that we can ensure their inclusion before publication.

Manuscript Click here to access/download;Manuscript;Chang Han.docx

Characterizing Nitrogen-Cycling Microorganisms and Genes in Sediments of the Three Gorges Reservoir

Chang Han[1], Zhiyuan Chen[1], Yihui Xiao[1], Ting Yang[2], Haoyang Shi[2], Huiqun Cao[2], Wenjun Yang[2, *]

1. College of Water Conservancy and Hydropower Engineering, Hohai University, Nanjing 210024, China

2. Changjiang River Scientific Research Institute, Wuhan 430010, China

Abstract: Microorganisms play a central role in driving the biogeochemical cycles in lakes (reservoirs). This study aims to refine the microbial-driven nitrogen cycle processes in the sediments of the Three Gorges Reservoir and assess the overall state of nitrogen cycling within these sediments. The study focuses on the Three Gorges Reservoir as the research area, systematically investigates the nitrogen cycle microorganisms and corresponding functional gene abundance characteristics attached to sediments from upstream, midstream, and downstream areas within the region, and explores key factors that may influence the composition of nitrogen cycle microbial communities. The primary findings of this paper include: 46 types of nitrogen conversion functional genes were annotated in the Three Gorges Reservoir sediment samples, covering seven major nitrogen cycle pathways: nitrogen fixation, nitrification, denitrification, nitrogen transport, organic nitrogen metabolism, assimilatory nitrate reduction, and dissimilatory nitrate reduction. The abundance of anaerobic ammonia oxidation functional genes is extremely low (almost zero), indicating that there may be no anaerobic ammonia oxidation potential on the particulate matter; there are differences in the abundance of nitrogen cycle functional genes and the composition of nitrogen cycle microbial communities among sediments with different particle sizes from upstream, midstream, and downstream areas, suggesting that sediment particle size may affect the composition of nitrogen cycle microbial communities attached to it; as sediment particle size decreases, the α and β diversity of nitrogen cycle microorganisms significantly increases, indicating that sediment particle size may have a significant impact on the diversity of nitrogen cycle microorganisms.

Keywords: Three Gorges Reservoir; Sediment; Nitrogen Cycle; Metagenomics

1 Introduction

Microorganisms involved in the nitrogen cycle play a primary role in driving the nitrogen cycle in lakes (reservoirs), influencing the generation and characteristics of various forms of nitrogen (Wan et al., 2017). They play a significant role in the biogeochemical cycling of nitrogen within the lake (reservoir) ecosystem (Xiong et al., 2023). The sediment in lakes (reservoirs) accumulates abundant nutrients and organic matter, which can provide food for nitrogen cycle microorganisms, attract microbial adhesion, and promote their growth and metabolism (Luef et al., 2009). Moreover, the sediment contains a strong gradient of environmental change, providing more ecological niches for nitrogen cycle microorganisms, leading to stronger functional

Author Introduction: Chang Han (1992-), E-mail: hanchang@hhu.edu.cn; *Corresponding Author, E-mail: yangwj@mail.crsri.cn

activity of nitrogen cycle-related microorganisms (Klawonn et al., 2015), influencing the nitrogen cycle process in lakes (reservoirs) (Zhu et al., 2013). The high density of microorganisms attached to the sediment surface facilitates interactions among them, facilitating the acquisition of functional traits, species differentiation, and evolution of nitrogen cycle microbial species (Steenackers et al., 2016; Bizic-Ionescu et al., 2018), leading to interactions among various processes of the nitrogen cycle (Xia et al., 2017). In recent years, the unique role of sediment in the nitrogen cycle process of lakes (reservoirs) has attracted the attention of many researchers.

Deposits from various regions and depths, due to their distinct compositions, origins, and redox conditions (Jia et al., 2016), exert a filtering effect on the species composition, community structure, and functional genes of nitrogen-cycling microorganisms (Bainbridge et al., 2012), leading to the occurrence of various nitrogen transformation processes or alterations in the intensity of nitrogen reaction processes on the deposits (Jia et al., 2016). However, current research on the nitrogen cycle processes driven by microorganisms attached to lake (reservoir) sediments often overlooks the impact of sediment particle size on the nitrogen cycle processes on particulates (Xia et al., 2017; Jia et al., 2016). Moreover, previous studies have primarily focused on a single nitrogen cycle process (Jia et al., 2016), with limited research on the overall nitrogen cycle process, resulting in researchers lacking a clear understanding of the complete nitrogen cycle process in lake (reservoir) sediments, neglecting potential interactions between different functional groups of microorganisms. This may lead to inaccurate assessments of the nitrogen cycle function in lake (reservoir) ecosystems.

Owing to the decrease in flow velocity along the Three Gorges Reservoir, there is a corresponding decline in hydrodynamic intensity, resulting in an overall downward trend in sediment particle size within the reservoir area. Data from the Hydrology Bureau of the Changjiang Water Resources Commission of the Ministry of Water Resources indicate a clear downstream decline in sediment particle size within the Three Gorges Reservoir. Specifically, the median particle size of sediments from Fengdu to the dam is approximately between 0.005-0.1 mm, while that of the pre-dam section is less than 0.01 mm. This results in a pronounced sediment particle size gradient from upstream to downstream within the Three Gorges Reservoir, providing excellent conditions for studying the nitrogen cycle process driven by microbial adhesion of sediments with different particle sizes. Therefore, this study collected nine sediment samples from Fengdu to the pre-dam section of the Three Gorges Reservoir, utilizing metagenomic element cycling techniques to investigate the functional gene composition of nitrogen transformation in the Three Gorges Reservoir sediments, microbial communities, and environmental influencing factors. This not only elucidates the mechanisms of nitrogen cycling within the Three Gorges Reservoir but also has significant implications for elucidating nitrogen cycling mechanisms in lakes (reservoirs), and can provide a theoretical basis for the prevention and control of nitrogen pollution in lakes (reservoirs).

2 Materials and Methods

2.1 Overview of the Study Area

The study area encompasses the Three Gorges Reservoir Region (Figure 1), a 20-county area impacted by the Three Gorges Project and designated for resettlement. Data from the Hydrology Bureau of the Changjiang Water Resources Commission of the Ministry of Water Resources indicate that sediment particle size in the reservoir region generally decreases downstream. The median particle size of sediments from Fengdu to the dam ranges from approximately 0.005 mm to 0.1 mm, with the median particle size of sediments before the dam being less than 0.01 mm. Consequently, nine sampling points were selected from Fengdu to the dam section and categorized as upstream, midstream, and downstream regions. The upstream sampling points include Xingyi Town, Fengdu County, Chongqing (S240), Wuyang Town, Zhong County, Chongqing (S219), and Zhongzhou Town, Zhong County, Chongqing (S206); midstream sampling points include Shuangjiang Street, Yunyang County, Chongqing (XJ001), Yongle Town, Fengjie County, Chongqing (S113), and Longmen Street, Wushan County, Chongqing (DN001); and downstream sampling points include Guizhou Town, Gui County, Yichang City, Hubei Province (XX001), Guizhou Town, Zigui County, Yichang City, Hubei Province (S39), and Maoping Town, Zigui County, Yichang City, Hubei Province (S32).

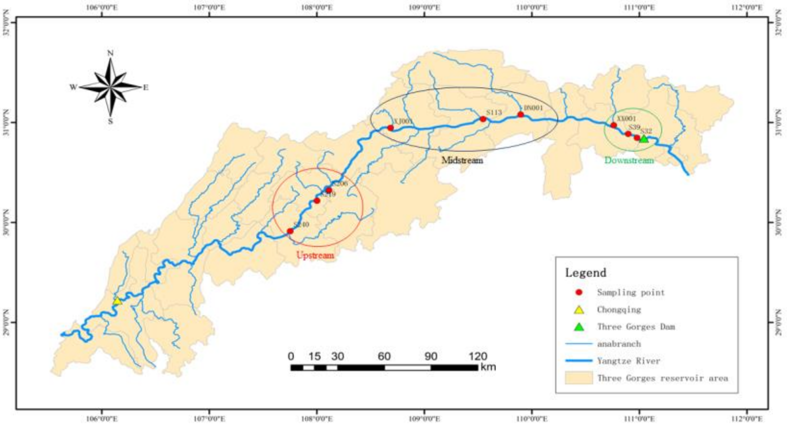

Figure 1 Schematic diagram of sampling points in the Three Gorges Reservoir

2.2 Sample Collection Method

Using a column-mounted sediment sampler, nine sampling sites were collected from 0-30 cm of sediment. Each sampling site was sampled with three tubes of sediment, and the sediment in each tube was divided into three equal portions, corresponding to depths of 0-10 cm, 10-20 cm, and 20-30 cm. The samples from the same depth in each tube were then uniformly mixed. Finally, all samples were placed in a 4°C storage box for preservation and promptly transported back to the laboratory. One portion of the samples was kept refrigerated at 4°C for determination of physicochemical properties; another portion was freeze-dried and then passed through a 100-mesh nylon sieve, and then stored frozen at -80°C for DNA extraction and molecular biology experiments.

2.3 Determination and Analysis of Physical and Chemical Properties

The physicochemical factor measurements were conducted in accordance with the standard testing method "Soil Agrochemical Analysis" (Bao, 2000). The moisture content (MC) and organic matter (OM) were determined using the loss of ignition (LOI) method (Zhang et al., 2007). The pH value was measured after adding ultrapure water to the sediment at a mass ratio of 5:1 of ultrapure water to sediment and leaching for 1 hour. The sediment samples were leached with a 2 $mol \cdot L^{-1}$ KCl solution (Yu et al., 2013), and the ammonia nitrogen (NH_4^+-N) and nitrate nitrogen (NO_3^--N) concentrations in the sediment were determined using a continuous flow analyzer (SAN plus, Skalar Analytical B.V., Breda, the Netherlands). The total carbon (TC) and total nitrogen (TN) were measured by burning the freeze-dried samples through a 100-mesh sieve, wrapped in aluminum foil, and then placed into an elemental analyzer (Elementar Vario EL III analyzer, Germany) for combustion determination. The total organic carbon (TOC) analyzer was used to automate the determination of dissolved organic carbon (DOC). All samples were subjected to three parallel measurements and the mean values were taken.

2.4 DNA Extraction and Metagenomic Sequencing

DNA was extracted from the samples using the fast DNA spin kit for soil DNA Kit (MP biomedicals, USA). In order to ensure the quality of extracted DNA, DNA quality was detected by 1% agarose gel electrophoresis, DNA concentration was detected by Quantus Fluorometer (PicoGreen), and DNA purity was detected by NanoDrop 2000. The results showed that high quality DNA was obtained. The DNA was submitted to Shanghai Majorbio Bio-pharm Technology Co.,Ltd. for quality inspection. Upon passing the inspection, the DNA was fragmented into fragments approximately 350 bp using the Covaris M220. Subsequently, a PE library was constructed, followed by bridge PCR, and finally, Illumina sequencing was performed on the sample.

2.5 Statistical analysis and quality control of sequencing data

In sequencing experiments, multiple samples are mixed together for parallel sequencing. Each sample is introduced with a sequence of Index tags that indicate its sample origin. Data from each sample is distinguished based on the Index sequence, and the extracted data is saved in fastq format. Quality assessment of the raw sequencing data from each sample is conducted, including statistics of base quality distribution and A/T/G/C base content distribution. The raw sequencing data is then subjected to quality control using the fastp software, yielding high-quality quality control data.

2.6 Sequence assembly and gene prediction

The assembly of sequences with varying sequencing depths was conducted using the Megahit software. Employing the succinct de Bruijn graph method, the assembly parameters were iteratively increased from small k-mers to larger k-mers, facilitating rapid sequence assembly. ORF predictions were performed on the contigs generated from the assembly results using the Prodigal software. Genes with nucleic acid lengths of no less than 100 bp were selected and translated into amino acid sequences, resulting

in a statistical table of gene prediction results for each sample.

2.7 Construction of genome sets and information statistics

The gene sequences predicted for all samples were clustered using CD-HIT software (default parameters: 90% sequence identity and 90% sequence coverage). The longest gene from each cluster was selected as the representative sequence, forming a non-redundant gene set. The high-quality reads from each sample were aligned to the non-redundant gene set using SOAPaligner software (default parameters: 95% identity), and the abundance information of genes in the corresponding samples was statistically analyzed.

2.8 Species and Functional Annotation

2.8.1 NR Species Annotation

Using BLASTP (BLAST Version 2.2.28+), the non-redundant gene set is aligned with the NR database. The species annotation results are obtained from the corresponding taxonomic information database of the NR library. Subsequently, the abundance of the species is calculated using the sum of gene abundance corresponding to the species. The abundance of species in various samples is statistically analyzed at eight taxonomic levels: Domain, Kingdom, Phylum, Class, Order, Family, Genus, and Species, thereby constructing an abundance profile at the corresponding taxonomic level.

2.8.2 KEGG functional annotation

The non-redundant gene set sequences were aligned with the gene database (GENES) of KEGG using BLASTP, with the expectation value (e-value) set at 1e-5 for the BLAST alignment parameters. Based on the alignment results, functional annotations were conducted using KOBAS 2.0 (KEGG Orthology Based Annotation System). The abundance of each functional category was calculated by summing the gene abundances corresponding to Orthology, Pathway, Enzyme, and Module.

2.9 Analysis of Genes Involved in the Nitrogen Cycle

Based on the nitrogen cycle gene set constructed from the nitrogen cycle KO numbers, the abundance information of genes in corresponding samples was statistically analyzed. The nitrogen cycle gene set was aligned with the NR database using BLASTP, resulting in species annotation. The sequence of the nitrogen cycle gene set was then aligned with the gene database of KEGG using BLASTP. Based on the alignment results, functional annotation was performed using KOBAS 2.0.

2.10 Data Analysis

In this study, we utilized R language for the creation of bar charts, pie charts, and heatmaps. We employed R language for Principal Component Analysis (PCA) and the generation of PCA diagrams. We utilized R language for Analysis of Molecular Variance (ANOSIM) and the production of box plots. We applied the Kruskal-Wallis

Rank Sum Test and created multiple group comparison diagrams using R language. We utilized LEfSe software for the generation of LEfSe diagrams. Based on KEGG annotation results, we conducted differential testing and visual analysis of enzymes with differential abundance in the nitrogen metabolism pathway. We employed iPath 3 for the creation of iPath metabolic pathway diagrams. Network analysis diagrams were created using Gehpi0.10.1. Relevant diagrams were prepared using Origin2024 and Microsoft Office 2021.

3 Results and Analysis

3.1 Analysis of Alpha Diversity

Alpha diversity, also known as within-habitat diversity, refers to the variety within a specific region or ecosystem. The alpha diversity index reflects the number of species and their relative abundance within a community, and is a result of competition or symbiosis among species for the same habitat. Comparing the alpha diversity indices of different samples reveals their diversity disparities. The ACE (Abundance-based Coverage Estimator) index is a method based on species richness, which takes into account the relative abundance of species. It is commonly used for estimating species richness, especially when the distribution of species richness is uneven. The Chao index is a non-parametric method for estimating species richness, which estimates the number of unobserved species based on the observed number of species and the number of rare species (i.e., species with only a single individual observed). As seen in Figures 2a and 2b, from the upstream to the downstream of the Three Gorges Reservoir, there is an increasing trend in the richness of nitrogen-cycling microorganisms with a decreasing trend in sediment particle size. Notably, significant differences are observed between the middle and upstream regions, as well as between the upstream and downstream regions. The Shannon index, which integrates information on both species richness and species evenness, is derived by calculating the negative logarithm of the sum of the relative abundance of each species (i.e., the proportion of individuals of that species in the total population). A higher value of the Shannon index indicates higher diversity and evenness among species in a community. The Shannon index gives equal weight to all species' relative abundance, making it equally sensitive to both rare and common species. The Simpson index mainly focuses on the abundance and dominance (i.e., proportion of certain species in a community) of species in a community. It is derived by calculating the reciprocal of the sum of the squares of all species' relative abundance. A smaller value of this index indicates higher diversity. The Simpson index is more sensitive to dominant species in a community, thus better reflecting uneven distribution of species in a community. As seen in Figures 2c and 2d, from the upstream to the downstream of the Three Gorges Reservoir, there is no significant change in the evenness of nitrogen-cycling microorganisms with a decreasing trend in sediment particle size, nor are there significant differences among the upstream, middle, and downstream regions.

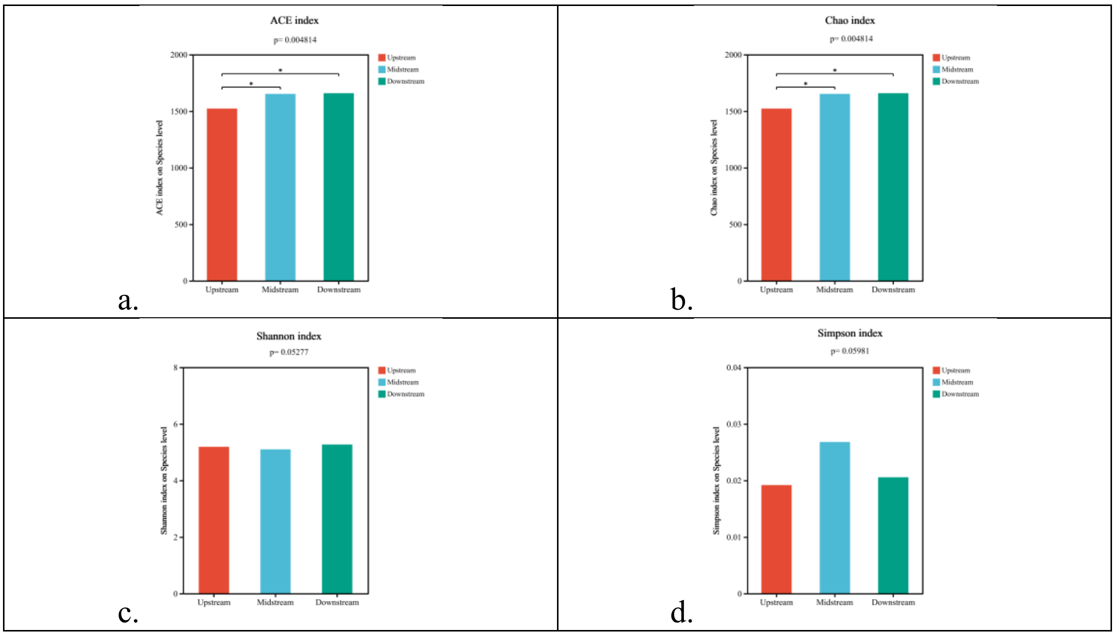

Figure 2 Presents the microbial α diversity indices of the nitrogen cycle in sediments from the Three Gorges Reservoir. (a) The ACE index; (b) The Chao index; (c) The Shannon index; and (d) The Simpson index.

3.2 Analysis of Species and Functional Composition

Utilizing KEGG data for functional gene annotation, a total of 46 nitrogen cycle functional genes were identified, encompassing eight nitrogen cycle functions: nitrogen fixation, nitrification, denitrification, anaerobic ammonia oxidation, nitrogen transport, organic nitrogen metabolism, assimilative nitrate reduction, and dissimilatory nitrate reduction. Notably, the relative abundance of genes for anaerobic ammonia oxidation is extremely low (nearly zero), indicating a significantly low metabolic potential for this process in the sediment, and potentially even the absence of anaerobic ammonia oxidation (Figure 3a). Upon comprehensive analysis of the relative abundance of microbial nitrogen cycle genes obtained from the annotation, it was observed that in the sediment upstream, midstream, and downstream of the Three Gorges Reservoir, the highest relative abundance of nitrogen cycle genes was observed in the organic nitrogen metabolism function, accounting for 46%-53% of all nitrogen cycle genes. This was followed by denitrification genes, which comprised 25%-26% of all functional genes in each group. The next most abundant categories were nitrogen transport genes (9%-14%), followed by dissimilatory nitrate reduction genes (6%-8%), assimilative nitrate reduction genes (2%-4%), nitrification genes (3%-3%), and finally nitrogen fixation genes (1%-2%).

From Figure 3a, it can also be observed that from the upstream to the midstream, the relative abundance of functional genes involved in organic nitrogen metabolism decreases, while the relative abundance of functional genes involved in nitrogen transport increases. Organic nitrogen metabolism involves the synthesis, decomposition, and transformation of organic nitrogen compounds, and is a key process in the cycling and utilization of nitrogen within organisms; whereas nitrogen transport refers to the movement and distribution of nitrogen within organisms and across ecosystems. The

decrease in the relative abundance of functional genes involved in organic nitrogen metabolism and the increase in the relative abundance of functional genes involved in nitrogen transport may indicate a diminished metabolic capacity of microorganisms for organic nitrogen, and an enhanced mobility of nitrogen within the ecosystem. This may be associated with a reduction in the available forms of organic nitrogen in sediments and an increase in inorganic forms of nitrogen. In addition to these two types of functional genes showing significant changes in relative abundance, the relative abundance of several other functional genes related to nitrogen cycling changes minimally from the upstream to the midstream. From the midstream to the downstream, there are only minor changes in the relative abundance of functional genes involved in organic nitrogen metabolism, nitrogen transport, assimilatory nitrate reduction, dissimilatory nitrate reduction, and nitrogen fixation, while there is no change in the relative abundance of functional genes involved in denitrification and nitrification.

As depicted in Figure 3b, when classifying microorganisms by their taxonomic groups, the highest relative abundance of microorganisms in the upper, middle, and lower reaches of the Three Gorges Reservoir is observed in the phylum Proteobacteria. The relative abundance of Proteobacteria is significantly higher than that of other microbial groups, thereby occupying an absolute dominant position. Proteobacteria is a very large bacterial phylum, classified as Gram-negative bacteria. These bacteria are distributed in various environments, including the ocean, soil, plants, animal hosts, and human microbiota. Moreover, Proteobacteria is capable of adapting to various extreme environmental conditions, such as deep-sea hydrothermal vents. Bacteria within the phylum Proteobacteria play diverse roles in the ecosystem, including nitrogen fixation, photosynthesis, and symbiotic relationships with hosts. They also contain some significant human pathogens. We can observe that the relative abundance of Proteobacteria increases from the upstream to the middle reaches, which may indicate changes in environmental and ecological processes. Some bacteria within Proteobacteria are involved in the nitrogen cycle, including nitrogen fixation, nitrification, and denitrification. Therefore, an increase in the abundance of Proteobacteria may suggest active nitrogen cycling in the sediments, which may be related to the availability and transformation rate of nitrogen in the environment. The relative abundance of Proteobacteria decreases from the middle to the lower reaches, which may indicate an impact on the nitrogen cycle, potentially leading to a reduction in available nitrogen in the ecosystem. However, on an overall comparison, the relative abundance of Proteobacteria in the lower reaches of the reservoir is still higher than that in the upstream sediments.

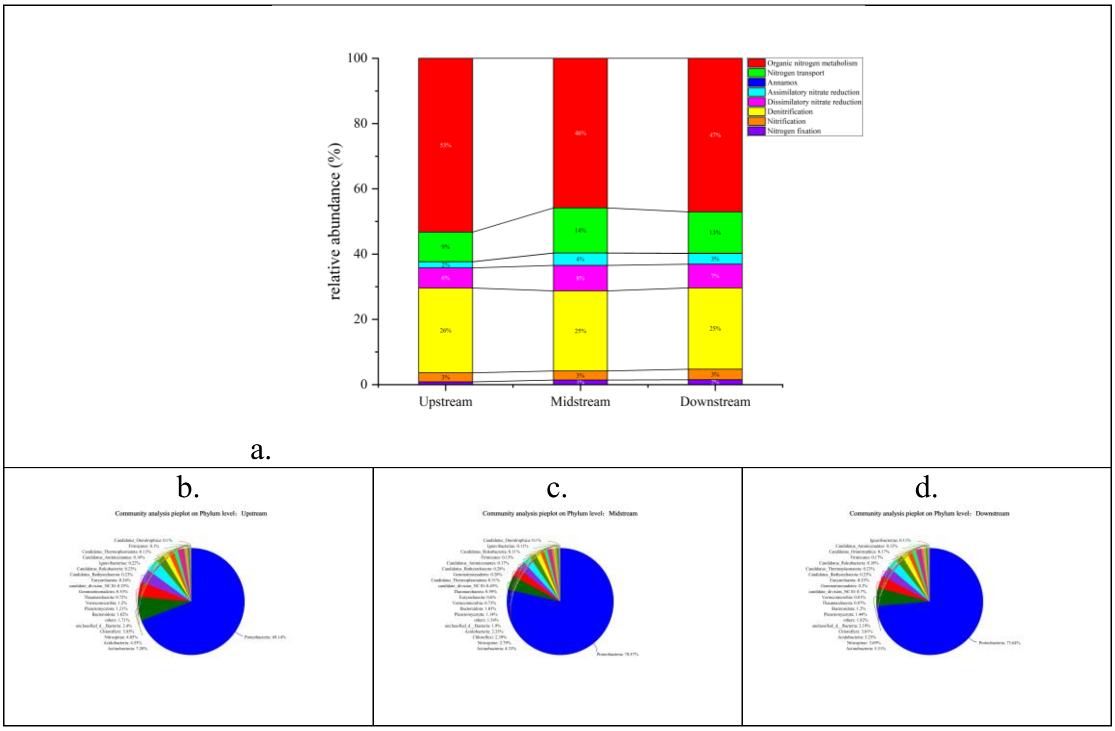

Figure 3 Presents an analysis of the microbial species and functional composition of the nitrogen cycle in the sediments of the Three Gorges Reservoir. (a) Presents a bar graph based on the functional gene level; (b) Presents a pie chart based on the phylum level (upstream); (c) Presents a pie chart based on the phylum level (midstream); and (d) Presents a pie chart based on the phylum level (downstream).

3.3 Comparative Analysis of Species and Function

As can be observed from Figure 4a, there is a clear upward trend in the Beta diversity of functional genes related to nitrogen cycling microorganisms in the sediment from upstream to downstream, suggesting that there may be an increasing trend in Beta diversity with decreasing particle size. As shown in Figure 4b, upstream samples exhibit a higher degree of aggregation and are further away from the midstream and downstream samples; midstream and downstream samples have a higher degree of dispersion, and the two samples are not clearly separated. As evident from Figure 4c, the inter-group differences are significantly greater than the intra-group differences, thus justifying the division into groups. Furthermore, as shown in Figure 4d, upstream, midstream, and downstream samples are classified at the species level into three groups. Among them, upstream samples cluster within the Betaproteobacteria_bacterium group, which is a specific bacterial species belonging to the Betaproteobacteria class, a class within the Proteobacteria phylum. The Betaproteobacteria class is a diverse group of bacteria found in various environments, including environmental microorganisms involved in biogeochemical processes such as nitrogen cycling in water bodies. Midstream and downstream samples are clustered within the Aquabacterium_sp. and Moraxellaceae_bacterium groups, with Aquabacterium_sp. belonging to a specific bacterial species of the Aquabacterium genus, a genus within the Betaproteobacteria class, which is typically found in freshwater environments. Moraxellaceae_bacterium is a specific bacterial species belonging to the Moraxellaceae family, a family under the

Pseudomonadales order and belonging to the Gammaproteobacteria class. Members of the Moraxellaceae family are widely distributed across various environments.

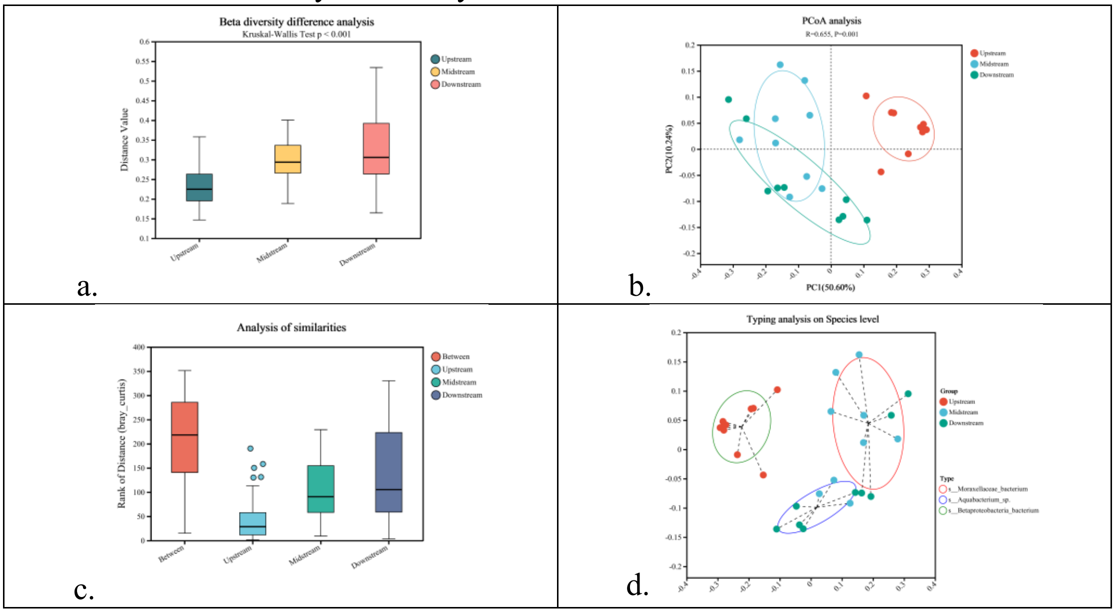

Figure 4 Presents a comparative analysis of microbial species and functions involved in the nitrogen cycle in the sediments of the Three Gorges Reservoir. (a) Analysis of inter-group differences in Beta diversity at the species level; (b) Principal Component Analysis (PCA) at the species level; (c) Analysis of Similarities at the species level; (d) Taxonomic analysis at the species level.

3.4 Analysis of Species and Functional Differences

The analysis of microbial communities using LEfSe reveals distinct species at the phylum to class level, which are visually represented in the upstream, midstream, and downstream regions. Nodes in the figure with different colors indicate microbial communities that are significantly enriched in their respective groups and have a significant impact on inter-group differences. Pale yellow nodes indicate microbial communities that do not exhibit significant differences across different groups or have no significant impact on inter-group differences. As can be observed from Figure 5a, only Fibrobacteres shows no significant differences among the three groups from upstream to downstream. The comparison of multiple groups is primarily used to analyze whether there are differences in microbial composition among the groups and to identify microorganisms with significant differences. These differentially represented microorganisms may be key features during environmental changes and can be utilized to search for microbial biomarkers across groups. Figure 5b shows the differences in average relative abundance of the same species across different groups, with differences being labeled. It visually depicts the significance of differences in the same species across multiple groups. At the species level, species with significant differences between upstream, midstream, and downstream include Moraxellaceae_bacterium,Betaproteobacteria_bacterium,Burkholderiales_bacterium, Aquabacterium_sp.,Gammaproteobacteria_bacterium,Burkholderiaceae_bacterium,Proteobacteria_bacterium,Acidobacteria_bacterium,Steroidobacteraceae_bacterium,Chloroflexi_bacterium,Actinomycetia_bacterium,uncultured_bacterium,Hyphomicrobiale

s_bacterium,Xanthomonadales_bacterium,and Alphaproteobacteria_bacterium.

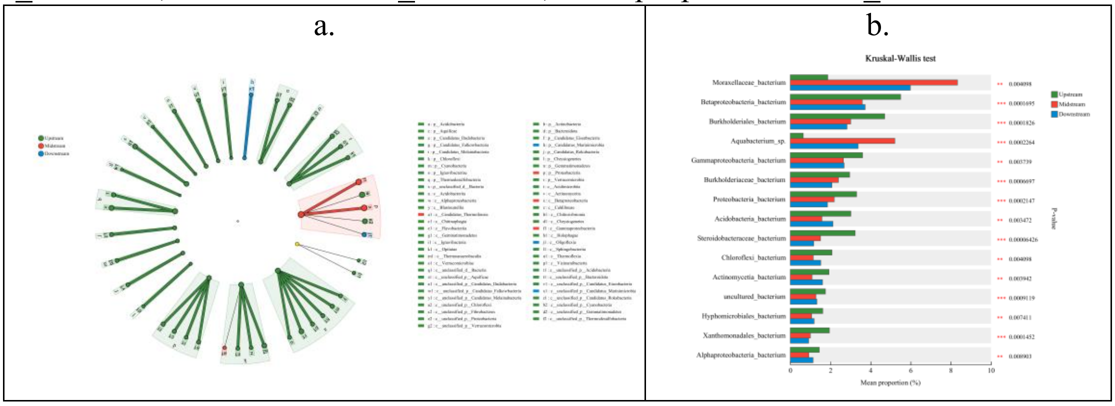

Figure 5 Presents an analysis of the species and functional differences in nitrogen cycling microorganisms within the sediments of the Three Gorges Reservoir. (a) Presents a LEfSe differential discrimination analysis conducted at the level of phyla to classes; (b) Presents a comparative analysis of multiple groups at the species level.

Based on the KEGG nitrogen metabolism pathway map, the relative abundance of enzymes in the pathway is marked. Boxes with filled colors in the map represent enzymes that have been labeled. The colors red and green indicate the relative abundance of enzymes in all samples, with red representing higher abundance and green representing lower abundance. As can be seen from Figure 6a, among all samples, the most abundant enzyme is EC 1.4.1.13, which is glutamate synthase. This enzyme plays a significant role in the nitrogen metabolism process of organisms.

Based on the results of KEGG annotations, differential tests and visualization analysis were conducted on enzymes with abundance differences in the nitrogen metabolism pathway. A significance analysis was performed on enzymes involved in this pathway across multiple groups. Bar charts were used to display the comparative differences in relative gene abundance of key enzymes in the metabolic pathway across different groups. In Figure 6b, G1, G2, and G3 represent the upstream, midstream, and downstream, respectively. Significance markers indicate significant differences between pairs of groups, while absence of a significance marker indicates no significant difference.

From Figure 6b, it is evident that the activity of EC 1.14.99.39, ammonia monooxygenase (AMO), significantly increases from upstream to downstream. AMO plays a pivotal role in the nitrogen metabolism process by converting ammonia (NH_3) into hydroxylamine (NH_2OH). This process represents the first step in the microbial oxidation of ammonia to nitrite (NO_2^-). The activity of EC 1.18.6.1, the enzyme classification code for nitrogenase, also significantly increases from upstream to downstream. Nitrogenase is an enzyme that converts atmospheric nitrogen (N_2) into NH_3, a process that is crucial for fixing atmospheric nitrogen into bioavailable forms. The activity of EC 1.18.6.2, the enzyme classification code for vanadium-dependent nitrogenase, also increases significantly from upstream to midstream, and then from midstream to downstream. This enzyme catalyzes the reduction of atmospheric N_2 into NH_3, which is crucial for biological nitrogen fixation. The activity of EC 1.4.1.4, the enzyme classification code for glutamate dehydrogenase, also increases significantly from upstream to midstream, and then from midstream to downstream. This enzyme

catalyzes the reaction between L-glutamate and water (H_2O) to produce 2-oxoglutarate, NH_3, reduced nicotinamide adenine dinucleotide phosphate (NADPH), and hydrogen ions (H^+). The activity of EC 1.7.1.15, the enzyme classification code for nitrite reductase (NADH), also increases significantly from upstream to midstream and then from midstream to downstream. This enzyme catalyzes the reaction between NH_3, H_2O, and three molecules of nicotinamide adenine dinucleotide (NAD^+), converting it into NO_2^-, three molecules of reduced nicotinamide adenine dinucleotide (NADH), and five hydrogen ions (H^+);The significant increase in EC 1.7.2.5 from upstream to downstream refers to the activity of nitric oxide reductase (cytochrome c), which catalyzes the reaction of nitrous acid (N_2O) and H_2O into nitric oxide (NO) and H^+. Simultaneously, it reduces two molecules of ferricytochrome c to ferrocytochrome c. The significant increase in EC 1.7.5.1 from upstream to downstream refers to the activity of nitrate reductase (quinone), which catalyzes the reaction of nitrate (NO_3^-) and quinone compounds (quinol) into NO_2^-, quinone, and H_2O. This reaction is crucial for the respiratory processes of certain bacteria under anoxic conditions.

The significance of EC 1.4.1.13 decreases from upstream to downstream. EC 1.4.1.13 is an enzymatic classification number representing glutamic acid synthase (NADPH), also known as glutamic acid (reduced nicotinamide adenine dinucleotide phosphate) synthase. This enzyme is involved in the nitrogen metabolism process, playing a key role in the synthesis of glutamic acid. The significance of EC 1.7.2.1 decreases from upstream to midstream and then increases from midstream to downstream. EC 1.7.2.1 refers to nitrite reductase (NO-forming), an enzyme that catalyzes the reaction of NO, H_2O, and iron-cytochrome C into NO_2^-, ferrocytochrome c, and two H^+. The significance of EC 1.7.2.2 decreases from upstream to downstream. EC 1.7.2.2 refers to nitrite reductase (cytochrome; ammonia-forming), an enzyme that plays a key role in the denitrification process of bacteria, catalyzing the reaction of NO_2^- into NH_3, while reducing an electron acceptor. The significance of EC 1.7.2.4 decreases from upstream to midstream and then increases from midstream to downstream. EC 1.7.2.4 refers to nitrous-oxide reductase, an enzyme that reduces N_2O into N_2. This enzyme plays a key role in the denitrification process of bacteria, especially in the last step of converting nitrous oxide into nitrogen gas. The significance of EC 1.7.7.2 decreases from upstream to midstream and then increases from midstream to downstream. EC1.7.7.2 refers to ferredoxin-nitrate reductase, an enzyme involved in nitrogen metabolism, catalyzing the reaction of NO_2^-, H_2O, and oxidized ferredoxin into NO_3^-, reduced ferredoxin, and two H^+. This reaction is very important in the nitrogen assimilation process of some microorganisms;The significance of EC 1.7.99.1 decreases significantly from upstream to downstream. EC 1.7.99.1 refers to hydroxylamine reductase, an enzyme capable of catalyzing the reduction of NH_2OH to NH_3 and H_2O. This enzyme plays an important role in certain bacteria, especially when dealing with hydroxylamine as a compound. The significance of EC 1.9.6.1 decreases significantly from upstream to downstream. EC 1.9.6.1 denotes cytochrome, an enzyme that catalyzes the reduction of NO_3^- to NO_2^- while oxidizing ferrocytochrome to ferricytochrome. This reaction is crucial for the respiratory processes of certain bacteria, especially under hypoxic conditions. The significance of EC 6.3.1.2 decreases

significantly from upstream to midstream and then to downstream. EC 6.3.1.2 is the enzyme classification number for glutamine synthetase, an enzyme that plays a central role in nitrogen metabolism. It catalyzes the synthesis of glutamine from glutamate and ammonia, while consuming ATP.

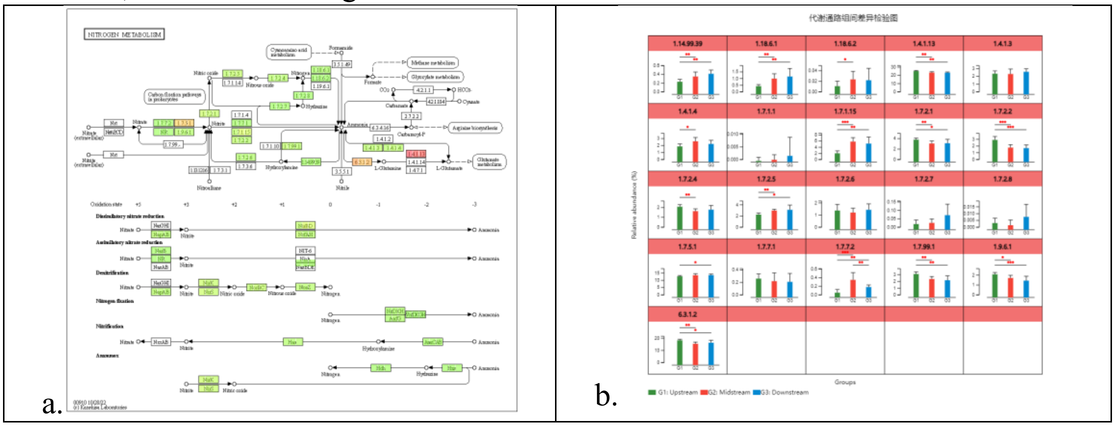

Figure 6 Presents an analysis of inter-group differences in nitrogen metabolic pathways in the sediments of the Three Gorges Reservoir. (a) Based on the nitrogen metabolic pathway diagram, the relative abundance differences of different group enzymes within the pathways are marked, visually demonstrating the activity of the same pathway across different groups; (b) A bar chart is used to compare the relative gene abundance of key enzymes in the metabolic pathways across different groups.

iPath 3 is a pathway diagram visualization tool that can be utilized for the analysis and customization of metabolic pathway diagrams. Through iPath 3, one can identify differences among complex metabolic pathways. As evident from Figure 7a, there are no differences in the nitrogen metabolism pathways between the upstream and midstream regions; Figure 7b shows that there are no differences between the midstream and downstream regions; and Figure 7c demonstrates that there are no differences between the upstream and downstream regions. Therefore, among the upstream, midstream, and downstream regions, the nitrogen metabolism pathways are consistent, with no differences in the metabolic pathways.

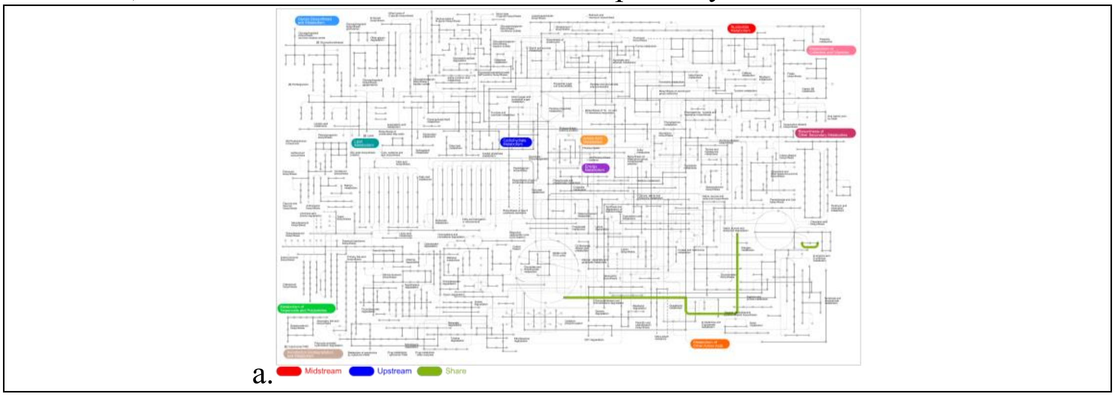

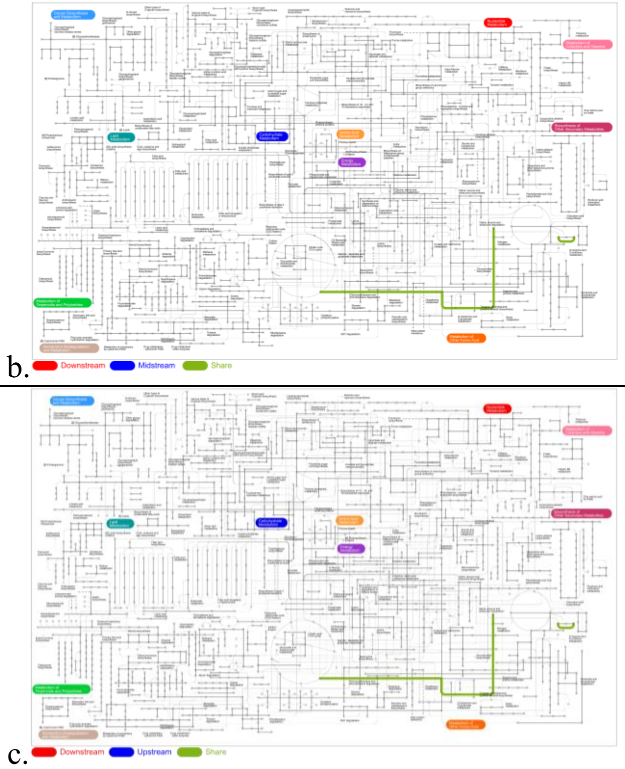

Figure 7 Depicts the ipath pathway diagram analysis. (a) The figure presents the overall ipath pathway diagram, wherein each line segment represents a distinct metabolic pathway. Among these, blue line segments indicate metabolic pathways unique to upstream samples, red line segments indicate metabolic pathways unique to midstream samples, and green line segments indicate shared metabolic pathways; (b) The figure presents the overall ipath pathway diagram, wherein each line segment represents a distinct metabolic pathway. Among these, blue line segments indicate metabolic pathways unique to midstream samples, red line segments indicate metabolic pathways unique to downstream samples, and green line segments indicate shared metabolic pathways; (c) The figure presents the overall ipath pathway diagram, wherein each line segment represents a distinct metabolic pathway. Among these, blue line segments indicate metabolic pathways unique to upstream samples, red line segments indicate metabolic pathways unique to downstream samples, and green line segments indicate shared metabolic pathways.

3.5 Correlative Analysis of Environmental Factors

The correlation heatmap illustrates the correlation between species and environmental factors, visually demonstrating the magnitude and significance of the correlations among various environmental factors and different species. Species are categorized at the phylum level. As evident from Figure 8a, there is a significant negative correlation between pH and Proteobacteria, unclassified_d_Bacteria, Bacteroidota, Verrucomicrobia, Gemmatimonadetes, and Ignavibacteriae; a significant negative correlation between NH_4^+ and Proteobacteria, Bacteroidota, and Gemmatimonadetes; a significant positive correlation between NO_3^- and Chloroflexi, Ignavibacteriae; a significant positive correlation between MC and Nitrospirae, Verrucomicrobia; a significant negative correlation between MC and

Candidatus_Bathyarchaeota, Candidatus_Thermoplasmatota, and Candidatus_Aminicenantes; a significant positive correlation between OM and Planctomycetota, Verrucomicrobia; and a significant negative correlation between OM and Candidatus_Bathyarchaeota. A significant positive correlation is observed between TN and Actinobacteria, Nitrospirae, Acidobacteria, Chloroflexi, unclassified_d__Bacteria, Bacteroidota, Verrucomicrobia, Gemmatimonadetes, and Ignavibacteriae; a significant positive correlation between TP and Nitrospirae, Bacteroidota, Verrucomicrobia, Gemmatimonadetes, Ignavibacteriae; and a significant negative correlation between TP and Euryarchaeota, Candidatus_Bathyarchaeota, Candidatus_Thermoplasmatota.

The Mantel Test network heatmap analysis involves conducting a correlation analysis between two matrices. In microbiomics, it is commonly utilized to assess the correlation between environmental factors and microbial community structure, categorizing species at the level of taxonomy. In Figure 8b, lines represent the correlation between communities and environmental factors, while the heatmap represents the correlation among environmental factors. From the figure, it is evident that the upstream is positively correlated with MC, OM, NO_3^-, and TP, with a significant positive correlation observed with MC. Additionally, there is a negative correlation with pH, NH_4^+, TN, and DOC. The midstream is positively correlated with pH, MC, OM, NH_4^+, NO_3^-, TN, TP, and DOC. The downstream is positively correlated with MC, OM, NH_4^+, TN, and DOC, while there is a negative correlation with pH, NO_3^-, and TP.

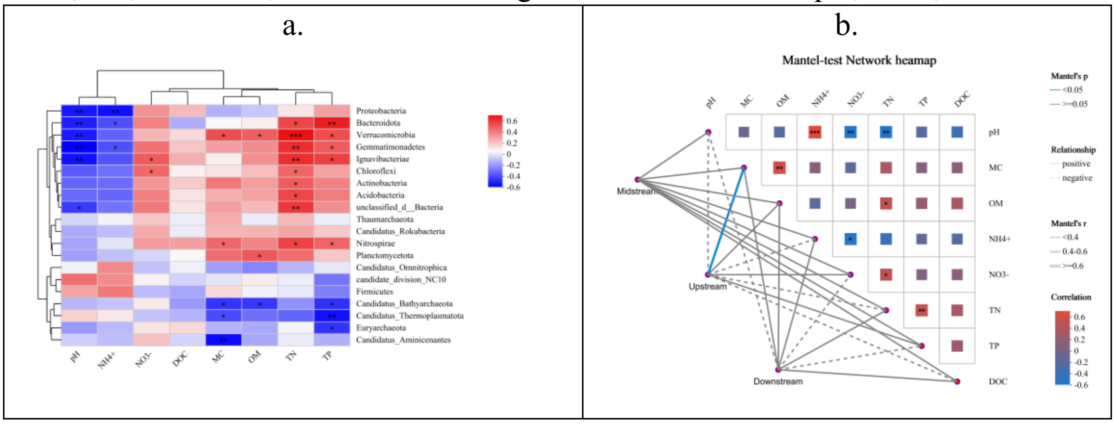

Figure 8 Presents the analysis of environmental factors. (a) Presents the correlation heatmap at the population level; (b) Presents the Mantel-test network heatmap at the species level.

3.6 Random Forest Analysis

Based on the random forest proximity matrix, a two-dimensional scatter plot (Figure 9a) was constructed. Green circles represent upstream samples, blue circles represent midstream samples, and red circles represent downstream samples. The scales on the x- and y-axes represent relative distance, which have no actual significance. Closer sample points indicate a more similar composition of species. As evident from the figure, the composition of species within the upstream, midstream, and downstream groups is more similar. The diagram (Figure 8b) based on random forest-derived flagship species and environmental factor importance rankings reflects the impact of

different species and environmental factors on the classification model's accuracy. In this diagram, the x-axis (Mean Decrease Accuracy) serves as a measure of importance, with higher values indicating greater significance. The y-axis shows the names of species and environmental factors arranged in order of importance. At the species level, flagship species include: Chloroflexi_bacterium_RBG_16_72_14, Betaproteobacteria_bacterium,Tepidicella_xavieri,Sphingorhabdus_pulchriflava,Nitrosomonas_sp.,Desulfurispirillum_indicum,uncultured_bacterium,Luteimonas_sp.,Leptothrix_sp._(in:_Bacteria),Nitratireductor_sp.,Hesseltinella_vesiculosa, Rubrivivax_sp., Sphingorhabdus_sp.,Proteobacteria_bacterium,Halioglobus_sp.,Candidatus_Aquabacterium_excrementipullorum,Desulfuromonas_sp.,Aquabacterium_olei,Lutibacter_maritimus. The flagship environmental factor is pH.

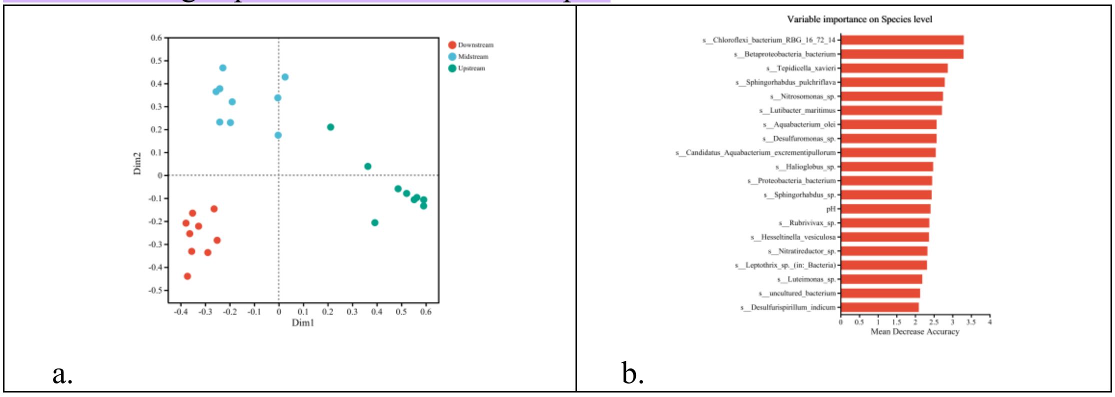

Figure 9 Presents the analysis of random forests. (a) Scatter plot reflecting the similarity between samples within and between groups based on the random forest proximity matrix; (b) Ranking plot of the importance of iconic species and environmental factors obtained from the random forest.

3.7 Network Analysis

Based on the level of nitrogen transformation functions, a network analysis diagram of the samples from the upstream, midstream, and downstream regions of the Three Gorges Reservoir is constructed. As evident from Figure 10a, among the samples from the upstream region, eight nitrogen transformation functions are clustered into six modules. The assimilation of nitrate reduction and nitrogen transport functions are clustered into one module, which accounts for 25% of the six modules. The organic nitrogen metabolism and denitrification functions are clustered into one module, which also accounts for 25% of the six modules. The nitrogen fixation, nitrification, anaerobic ammonia oxidation, and dissimilatory nitrate reduction functions are each clustered independently into one module, each accounting for 12.5% of the six modules. The diagram shows that positive correlations account for 100% of the lines, indicating that all eight nitrogen transformation functions in the upstream samples are positively correlated with each other. From Figure 10b, it is evident that in the midstream samples, eight nitrogen transformation functions are clustered into four modules. The assimilation of nitrate reduction, nitrogen transport, and dissimilatory nitrate reduction functions are clustered into one module, which accounts for 37.5% of the four modules. The nitrogen fixation, organic nitrogen metabolism, and denitrification functions are clustered into one module, which also accounts for 37.5% of the four modules. The

nitrification and anaerobic ammonia oxidation functions are each clustered independently into one module, each accounting for 12.5% of the four modules. The diagram shows that positive correlations account for 68.75% of the lines and negative correlations account for 31.25%, indicating that in the midstream samples, positive correlations dominate among the eight nitrogen transformation functions. As evident from Figure 10c, eight nitrogen transformation functions are clustered into two modules. The nitrification, organic nitrogen metabolism, denitrification, and anaerobic ammonia oxidation functions are clustered into one module, which accounts for 50% of the two modules. The nitrogen transport, nitrogen fixation, assimilation of nitrate reduction, and dissimilatory nitrate reduction functions are clustered into one module, which also accounts for 50% of the two modules. The diagram shows that positive correlations account for 60% of the lines and negative correlations account for 40%, indicating that in the downstream samples, positive correlations among the eight nitrogen transformation functions still dominate.

Based on the species-level analysis of the upstream, midstream, and downstream samples of the Three Gorges Reservoir, a network analysis diagram was constructed. As shown in Figure 10d, among the upstream samples, nitrogen-cycling microorganisms were clustered into 15 modules based on species level classification. The highest proportion module accounted for 33.33% of all modules, followed by the second-highest proportion module accounting for 25.81%, the third-highest proportion module accounting for 16.13%, the fourth-highest proportion module accounting for 6.45%, and the fifth and sixth-highest proportion modules each accounting for 3.23%. The proportion of modules ranging from the seventh to the fifteenth accounted for 1.08% of all modules. In the figure, positively correlated links accounted for 79.22%, while negatively correlated links accounted for 20.78%, indicating that positive correlations among nitrogen-cycling microorganisms dominate in the upstream samples. As shown in Figure 10e, among the midstream samples, nitrogen-cycling microorganisms were clustered into 17 modules based on species level classification. The highest proportion module accounted for 29.03% of all modules, followed by the second-highest proportion module accounting for 19.35%, the third-highest proportion module accounting for 12.9%, the fourth-highest proportion module accounting for 10.75%, the fifth-highest proportion module accounting for 7.53%, the sixth and seventh-highest proportion modules each accounting for 4.3%, the eighth-highest proportion module accounting for 2.15%, and modules ranging from the ninth to the seventeenth each accounting for 1.08%. In this figure, positively correlated links accounted for 81.12%, while negatively correlated links accounted for 18.88%, indicating that positive correlations among nitrogen-cycling microorganisms dominate in the midstream samples.As depicted in Figure 10f, within the downstream samples, the nitrogen cycle microorganisms are clustered into 16 modules based on their horizontal classification. The module with the highest proportion accounts for 40.4% of all modules, followed by a module accounting for 33.33%, with the third to fifth modules each accounting for 3.03%, and the sixth to eleventh modules each accounting for 2.02%. The modules from twelfth to sixteenth each account for 1.01%. In the figure, positively correlated connections account for 60.46%, while negatively correlated connections account for

39.54%. This suggests that in the midstream samples, the nitrogen cycle microorganisms primarily exhibit positive correlations among themselves.

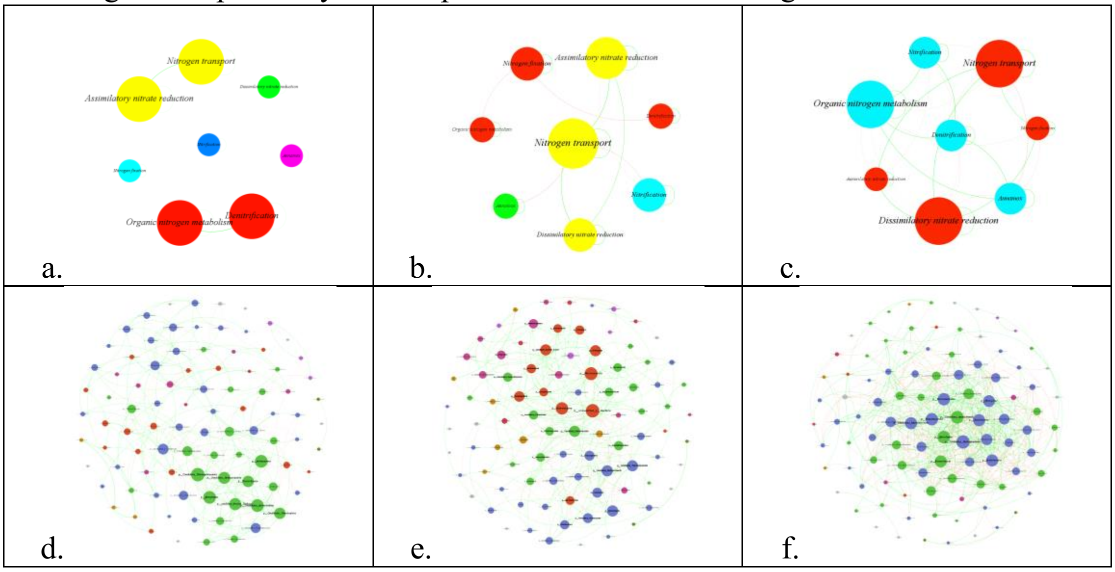

Figure 10 Presents the network analysis diagrams. (a) is based on the functional gene level, referred to as the upstream region; (b) is based on the functional gene level, referred to as the midstream region; (c) is based on the functional gene level, referred to as the downstream region; (d) is based on the phylum level, referred to as the upstream region; (e) is based on the phylum level, referred to as the midstream region; (f) is based on the phylum level, referred to as the downstream region.

4 Discussion

Based on the metagenomic sequencing results of the sediment from the Three Gorges Reservoir, seven nitrogen cycle functional genes—nitrogen fixation, nitrification, denitrification, nitrogen transport, organic nitrogen metabolism, assimilatory nitrate reduction, and dissimilatory nitrate reduction—were detected from the reservoir's upstream to downstream. However, the relative abundance of the anaerobic ammonia oxidation gene was extremely low (almost zero) in all samples, indicating that the metabolic potential for anaerobic ammonia oxidation in the reservoir sediment is extremely low, or even nonexistent. Anaerobic ammonia oxidation requires specific environmental conditions such as anoxic conditions, suitable temperature, and pH, and the absence of such processes in the reservoir may reflect the absence of certain specific conditions. Further exploration into which specific conditions are lacking is necessary in subsequent experiments. The greenhouse gas emissions generated during anaerobic ammonia oxidation are relatively low; the absence of this process may imply an increase in other nitrogen removal processes (such as denitrification) (Peng et al., 2018; Li, 2020). According to the results of this study, the relative abundance of denitrification functional genes in the reservoir's upstream, midstream, and downstream sediment is above 25%, indicating that denitrification is the primary nitrogen removal process in the Three Gorges Reservoir (Zhang, 2018; Yang et al., 2023). Denitrification may produce and emit N_2O, a greenhouse gas, during its process, while anaerobic ammonia oxidation produces almost no N_2O (Sheng et al., 2016). This suggests that the Three Gorges Reservoir has a high potential for N_2O emissions. As a greenhouse gas,

N_2O has a global warming potential much higher than that of CO_2, indicating that the Three Gorges Reservoir may be a significant source of greenhouse gas emissions.

This study indicates that variations in sediment particle size may have an impact on the functional gene composition of nitrogen cycling microorganisms. From upstream to midstream, as the sediment particle size gradually decreases, the relative abundance of functional genes related to organic nitrogen metabolism decreases. This suggests that larger sediment particles may possess a higher potential for organic nitrogen metabolism. Organic nitrogen metabolism is a pivotal process in the nitrogen cycle in aquatic environments. Numerous previous studies have demonstrated that larger particle sizes have higher nitrogen metabolic activity compared to smaller particle sizes (Hsiao et al., 2014; Zhang et al., 2014; Hou et al., 2018; Li, 2020; Zhao et al., 2020). This may be attributed to the fact that larger sediment particles have a greater concentration of organic nitrogen (Filho et al., 2011; Sanderson et al., 2011; Yao et al., 2023), providing a sufficient organic nitrogen source for microorganisms, leading to a higher relative abundance of functional genes related to organic nitrogen metabolism on larger sediment particles. Additionally, from the upstream to midstream of the reservoir, as the sediment particle size gradually decreases, the relative abundance of functional genes related to nitrogen transport increases. This increase in nitrogen transport genes may reflect an enhanced capability of microorganisms to utilize ammonia-nitrogen (NH_4^+), nitrite-nitrogen (NO_2^-), and nitrate-nitrogen (NO_3^-) in sediments (Dai et al., 2013). This change in gene abundance may indicate a shift in microbial community structure in sediments, potentially due to the reduction in organic nitrogen attached to the sediment particles as particle size decreases. Therefore, microbial efficiency in utilizing organic nitrogen decreases, while their efficiency in converting inorganic nitrogen increases. Thus, sediment particle size may influence the abundance of functional genes related to nitrogen cycling microorganisms attached to it.

Based on the functional genes related to the nitrogen cycle identified through metagenomic analysis, the taxonomic annotation of the corresponding species reveals that there are differences in the composition of microbial communities driving the various reactions of the nitrogen cycle upstream, midstream, and downstream of the reservoir. This study found that at the phylum level, the dominant microorganisms driving the nitrogen cycle upstream, midstream, and downstream are all Proteobacteria, and Proteobacteria have a significant abundance advantage over other phyla. The relative abundance of Proteobacteria in the sediments of the Three Gorges Reservoir is significantly higher than that of other phyla, which may indicate several points: ① ecological diversity, Proteobacteria contain a variety of ecological types of bacteria, such as photosynthetic, organic matter decomposition, pathogenic, and symbiotic bacteria, among others. Their high abundance may reflect the diversity of ecological niches and active biogeochemical processes in the sediments (Emenike et al., 2023); ② environmental adaptability, Proteobacteria have broad environmental adaptability and can survive under various environmental conditions, including extreme pH, temperature, and salinity, which may be one of the reasons for their dominance in sediments (Miller et al., 2023); ③ participation in the nitrogen cycle, some bacteria in

Proteobacteria, such as ammonia-oxidizing bacteria (AOB) and nitrate-reducing bacteria, play key roles in the nitrogen cycle. Their high abundance may indicate active nitrogen transformation processes in the sediments (Hou et al., 2024); ④ organic matter processing, bacteria in Proteobacteria play an important role in the decomposition of organic matter and nutrient cycling. The rich organic matter in sediments may promote the growth of these bacteria (Liu et al., 2024); ⑤ pathogenicity and symbiosis, some Proteobacteria are symbiotic with animals and plants or have pathogenicity. Their high abundance in sediments may be related to the diversity and abundance of their host organisms (Zhang et al., 2024). In summary, the high abundance of Proteobacteria in sediments may be related to their extensive ecological functions, environmental adaptability, role in the nitrogen cycle, organic matter processing capacity, and interactions with other organisms. These characteristics make Proteobacteria an important player in the sediment ecosystem.

From the upstream to the downstream of the Three Gorges Reservoir, as the grain size of sediments decreases along the flow path, there is a noticeable increase in the diversity of nitrogen cycling microorganisms. This trend may be attributed to the following factors: smaller sediment grain sizes provide a larger specific surface area, offering more adhesion sites for microorganisms, thereby supporting a greater number of microbial communities (Qin et al., 2022; Daniel et al., 2023; Sebego et al., 2023); fine sediment particles can better retain moisture and nutrients, providing a rich source of nutrition for microorganisms, thus promoting an increase in microbial diversity (Burrascano et al., 2013; Domangue et al., 2018); small grain sizes can create more micro-environments, such as tiny fissures and pores, which provide suitable habitats for different types of microorganisms (Peterson, 2019; Douglas et al., 2021); the lower permeability of fine sediment particles may limit the diffusion of oxygen, thereby creating varying redox conditions in the sediments, supporting a more diverse microbial community (Liu et al., 2021); the surface of fine sediment particles can adsorb more organic matter and nutrient salts, providing essential nutrients for microorganisms and promoting their growth and reproduction (Dafa-Alla et al., 2024); fine sediment particles are generally more stable and less susceptible to water flow disturbances, offering a relatively stable and continuous living environment for microorganisms (Kozono et al., 2009). It is likely that the combined effects of these factors enable fine sediment particles to support a more diverse microbial community. From the upstream to the downstream of the Three Gorges Reservoir, as the grain size of sediments decreases along the flow path, there is an increasing trend in sample dispersion within the nitrogen cycling microbial community. This trend may be attributed to the following factors: smaller sediment grain sizes may exhibit higher environmental heterogeneity because they can create more microspaces and different redox conditions, thereby supporting microbial communities with different ecological niches (Chen et al., 2010; GuimaresDurán et al., 2024); the larger specific surface area of fine sediment particles may enhance the adsorption and retention of nutrients, leading to uneven distribution of nutrients, thereby promoting the differentiation of microbial communities (André et al., 2024);The small-sized sediment particles may offer better physical protection to microorganisms, reducing predation pressure and mechanical disturbances. Moreover,

bioturbation may create more localized ecological niches in fine-grained sediments, enhancing the diversity of microbial communities (Jarcuska et al., 2024). Fine-grained sediments may be less affected by hydrodynamic forces, leading to a more uneven distribution of water and nutrients within the sediments, thereby influencing the composition of microbial communities (Mathieu et al., 2024). The sediment-water interface of fine-grained sediments may be more complex, involving a greater number of biogeochemical processes that may promote the differentiation of microbial communities (Sakir et al., 2024). In fine-grained sediments, due to higher environmental heterogeneity, interactions among microorganisms (such as competition and symbiosis) may become more intricate, leading to increased differences in microbial community structure (Kim et al., 2024).

This study indicates that the nitrogen cycling process on the sediments of the Three Gorges Reservoir may be influenced by the size of the sediment particles. There are differences in the functional gene composition of nitrogen cycling microorganisms attached to sediments of varying particle sizes. Sediments are currently a research hotspot in the field of water nitrogen cycling, representing a novel approach to address water eutrophication. However, since the various processes of nitrogen transformation are not entirely independent, they may exhibit coupled processes under certain conditions, such as the nitrification-denitrification coupling promoting nitrogen removal from water bodies. Therefore, further exploration of the nitrogen cycling process on sediments is still necessary.

5 Conclusion

This study focuses on the Three Gorges Reservoir as the research area and employs metagenomic sequencing technology to systematically identify the nitrogen cycle microorganisms and corresponding functional gene abundance characteristics associated with nitrogen cycling on the sediments of the upstream, midstream, and downstream regions of the study area. The main conclusions are as follows: (1) A total of 46 nitrogen conversion functional genes have been annotated in the sediment samples of the Three Gorges Reservoir, covering seven major nitrogen cycle pathways: nitrogen fixation, nitrification, denitrification, nitrogen transport, organic nitrogen metabolism, assimilative nitrate reduction, and dissimilative nitrate reduction, indicating that there are potential nitrogen cycle capabilities on the sediments of the Three Gorges Reservoir. The abundance of anaerobic ammonia oxidation functional genes is extremely low (almost zero), suggesting that there may be no anaerobic ammonia oxidation potential on the particulate matter; (2) There are differences in the abundance of nitrogen cycle functional genes and the community composition of nitrogen cycle microorganisms among sediments of different particle sizes in the upstream, midstream, and downstream regions, indicating that the size of the sediment particles may affect the community composition of nitrogen cycle microorganisms attached to them; (3) With the reduction in sediment particle size, the α and β diversity of nitrogen cycle microorganisms significantly increase, indicating that the size of sediment particles may have a significant impact on the diversity of nitrogen cycle microorganisms.

References:

André Felipe Araujo Lira, Moura G J B, Stênio talo Araújo Foerster. Scorpion assemblages in threatened Brazilian forests: The role of environmental factors in explaining beta-diversity patterns[J]. Insect conservation and diversity, 2024(1): 17.

Bainbridge Z T, Wolanski E, Álvarez-Romero J G, et al. Fine sediment and nutrient dynamics related to particle size and floc formation in a Burdekin River flood plume, Australia[J]. Marine Pollution Bulletin, 2012, 65: 236-248.

Bao Shidan. Soil Agrochemical Analysis (3rd Edition)[M]. China Agricultural Publishing House, 2000.

Bizic-Ionescu M, Ionescu D, Grossart H P. Organic Particles: Heterogeneous hubs for microbial interactions in aquatic ecosystems[J]. Frontiers in Microbiology, 2018, 9: 2569.

Burrascano S, Keeton W S, Sabatini F M, et al. Commonality and variability in the structural attributes of moist temperate old-growth forests: A global review[J]. Forest Ecology and Management, 2013, 291: 458-479.

Dafa-Alla D A M, Gibreel H H, Siddig A A H, et al. Exploring Trees Diversity and Ecological Value of Mountain Forests in the Blue Nile Region of Sudan[J]. Open Journal of Ecology, 2024, 14(3): 12.

Dai J, Gao G, Chen D, et al. Effects of trophic status and temperature on communities of sedimentary ammonia oxidizers in Lake Taihu[J]. Geomicrobiology Journal, 2013, 30: 886-896.

Daniel H, Heriniaina R, Mahefa A N N, et al. Forest fragmentation and its associated edge-effects reduce tree species diversity, size, and structural diversity in Madagascar's transitional forests[J]. Biodiversity and Conservation, 2023(10): 32.

Domangue R J, Mortazavi B. Nitrate reduction pathways in the presence of excess nitrogen in a shallow eutrophic estuary[J]. Environmental Pollution, 2018, 238: 599-606.

Douglas A R, Murgulet D, Montagna P A. Hydroclimatic variability drives submarine groundwater discharge and nutrient fluxes in an anthropogenically disturbed, semi-arid estuary[J]. Science of the Total Environment, 2021, 755: 142574.

Emenike C U, Agamuthu P, Fauziah S H, et al. Enhanced Bioremediation of Metal-Contaminated Soil by Consortia of Proteobacteria[J]. Water, air and soil pollution, 2023(12): 234.

GuimaresDurán Carolina Leite, Miranda L T F, Ramos M B, et al. Upstream cascade reservoirs drive temporal beta diversity increases through species loss in a dammed river[J]. FEMS Microbiology Ecology, 2024(1): 1.

Hou L, Xie X B, Wan X H, et al. Niche differentiation of ammonia and nitrite oxidizers along a salinity gradient from the Pearl River estuary to the South China Sea[J]. Biogeosciences, 2018, 15: 5169-5187.

Hou M, Zhu Y, Chen H. Chiral herbicide imazethapy influences plant-soil feedback on nitrogen metabolism by shaping rhizosphere microorganisms[J]. Environmental Science and Pollution Research, 2024(12): 31.

Hsiao S S Y, Hsu T C, Liu J. W, et al. Nitrification and its oxygen consumption along the turbid Chang Jiang River plume[J]. Biogeosciences, 2014, 11: 2083-2098.

Jarcuska B, Galffyova M, Schnuermacher R, et al. Solar parks can enhance bird diversity in agricultural landscape[J]. Journal of Environmental Management, 2024(Feb.1): 351.

Jia Z M, Xia T, Xia X H, et al. Effect of particle size and composition of suspended sediment on denitrification in river water[J]. Science of The Total Environment, 2016, 541, 934-940.

Junyi Zhang. Metagenomic Studies On Cyanobacterial Blooms In Lake Taihu[D]. Southeast University, 2018.

Kim Y, Ban G H, Hong Y W, et al. Bacterial profile of pork from production to retail based on high-throughput sequencing[J]. Food Research International, 2024(Jan.): 176.

Klawonn I, Bonaglia S, Brüchert V, et al. Aerobic and anaerobic nitrogen transformation processes in N_2-fixing cyanobacterial aggregates[J]. The ISME Journal, 2015, 9: 1456-1466.

Kozono T, Koyaguchi T. Effects of relative motion between gas and liquid on 1-dimensional steady flow in silicic volcanic conduits: 1. An analytical method[J]. Journal of Volcanology & Geothermal Research, 2009, 180(1): 21-36.

Liu S, Zhang P, Wang X, et al. Comparative analysis of different bio-organic fertilizers on growth and rhizosphere environment of grapevine seedlings[J]. Scientia horticulturae, 2024: 324.

Liu Y X, Qin Y, Chen T, et al. A practical guide to amplicon and metagenomic analysis of microbiome data[J]. Proteins and Cells, 2021, 12(5): 16.

Luef B, Neu T R, Peduzzi P. Imaging and quantifying virus fluorescence signals on aquatic aggregates: A new method and its implication for aquatic microbial ecology[J]. FEMS Microbiology Ecology, 2009, 68: 372-380.

Mathieu H, Jacques G, Jardim V L, et al. Changes in maerl-associated macroalgal community dynamics as evidence of anthropogenic pressure[J]. Annals of Botany, 2024.

Miller S E, Colman A S, Waldbauer J R. Metaproteomics reveals functional partitioning and vegetational variation among permafrost-affected Arctic soil bacterial communities[J]. mSystems, 2023, 8(3).DOI:10.1128/msystems.01238-22.

Peng Yu-ke, Lu Jun-ling, Chen Hui-ping, et al. Dynamic Changes of Nitrogen-Transforming and Phosphorus-Accumulating Bacteria Along with the Formation of Cyanobacterial Blooms[J]. Environmental Science, 2018, 39(11): 4938-4945.

Peterson C J. Damage Diversity as a Metric of Structural Complexity after Forest Wind Disturbance[J]. Forests, 2019, 10(2): 85.

Qin Y, He X, Lei X, et al. Tree size inequality and competition effects on nonlinear mixed effects crown width model for natural spruce-fir-broadleaf mixed forest in northeast China[J]. Forest Ecology and Management, 2022.

Sakir A, Soumendu M, Rasmita M, et al. 16s RNA-based metagenomics reveal previously unreported gut microbiota associated with reactive arthritis and undifferentiated peripheral spondyloarthritis[J]. Rheumatology, 2024.

Sebego R, Paul André Ebanga, Fobane J L, et al. Environmental, structural, and taxonomic diversity factors drive aboveground carbon stocks in semi-deciduous

tropical rainforest strata in Cameroon[J]. African Journal of Ecology, 2023, 61(1): 163-175.
Sheng P, Yu Y, Zhang J, et al. Bacterial diversity and distribution in seven different estuarine sediments of Poyang Lake, China[J]. Environmental Earth Science, 2016, 75: 479.
Shengbin Chen, Zhiyun O, Weihua X, et al. A review of beta diversity studies[J]. Biodiversity Science, 2010, 18(4) :323-335.
Steenackers H P, Parijs I, Foster K R, et al. Experimental evolution in biofilm populations[J]. FEMS Microbiology Reviews, 2016, 40: 373-397.
Wan Y, Ruan X H, Zhang Y P, et al. Illumina sequencing-based analysis of sediment bacteria community in different trophic status freshwater lakes[J]. Microbiology Open, 2017, 6: e00450.
Xia X H, Liu T, Yang Z F, et al. Enhanced nitrogen loss from rivers through coupled nitrification denitrification caused by suspended sediment[J]. Science of The Total Environment, 2017, 579: 47-59.
Xiong G Y, Zhu X B, Wu J C, et al. Seawater intrusion alters nitrogen cycling patterns through hydrodynamic behavior and biochemical reactions: Based on Bayesian isotope mixing model and microbial functional network[J]. Science of The Total Environment, 2023, 867, 161368.
Yang Xiaolong, Liu Lihua, Bi Yonghong, et al. Progress on photosynthetic nitrogen assimilation and its regulatory mechanisms in cyanobacteria[J]. Lake Science, 2023, 35(3): 766-779.
Yu Xianghua, Zhang Ming. Study on the determination conditions of ammonium nitrogen content in soil[J]. Agricultural Technology & Equipment, 2013, 225 (3): 11-12.
Yufang Li. Size-fractionated nitrification driven by marine microorganisms[D]. Xiamen University, 2020.
Zhang J, Liu K, Li Y, et al. Characterization and seasonal variation in biofilms attached to leaves of submerged plant[J]. World Journal of Microbiology & Biotechnology, 2024(1): 40.
Zhang Wenhe, Mu guijin. Accuracy control of determination of organic matter and carbonate by loss on ignition method[J]. Arid Land Geography, 2007, 30 (3): 137-141.
Zhang Y, Xie X, Jiao N, et al. Diversity and distribution of amoA-type nitrifying and nirS-type denitrifying microbial communities in the Yangtze River estuary[J]. Biogeosciences, 2014, 11: 2131-2145.
Zhao Z H, Baltar F, Herndl G J. Linking extracellular enzymes to phylogeny indicates a predominantly particle-associated lifestyle of deep-sea prokaryotes[J]. Science Advances, 2020, 6: eaaz4354.
Zhu G B, Wang S Y, Wang W D, et al. Hotspots of anaerobic ammonium oxidation at land–freshwater interfaces[J]. Nature Geoscience, 2013, 6: 103-107.

Figures

Figure 1:

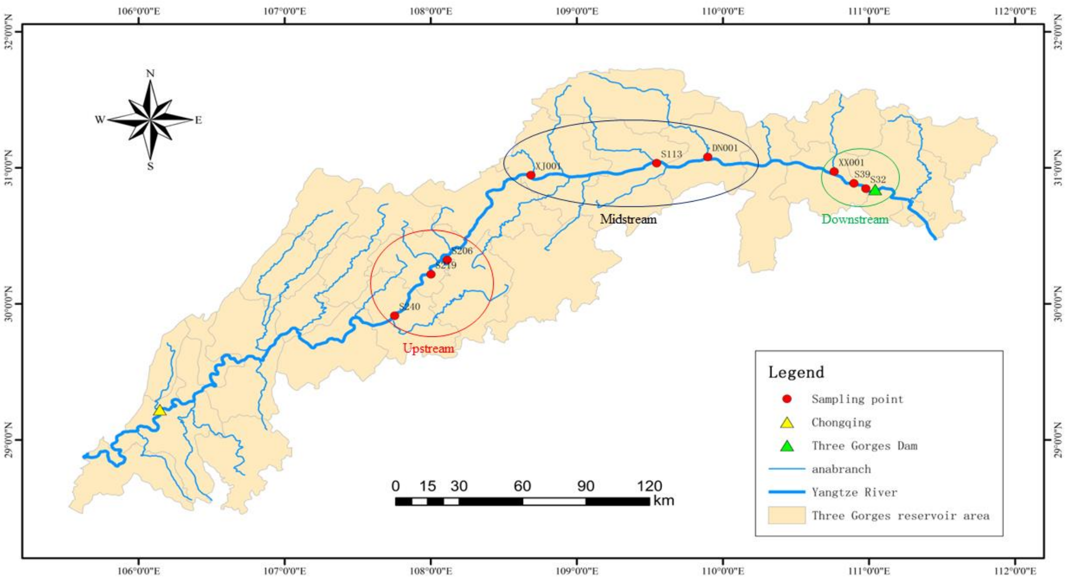

Figure 2a:

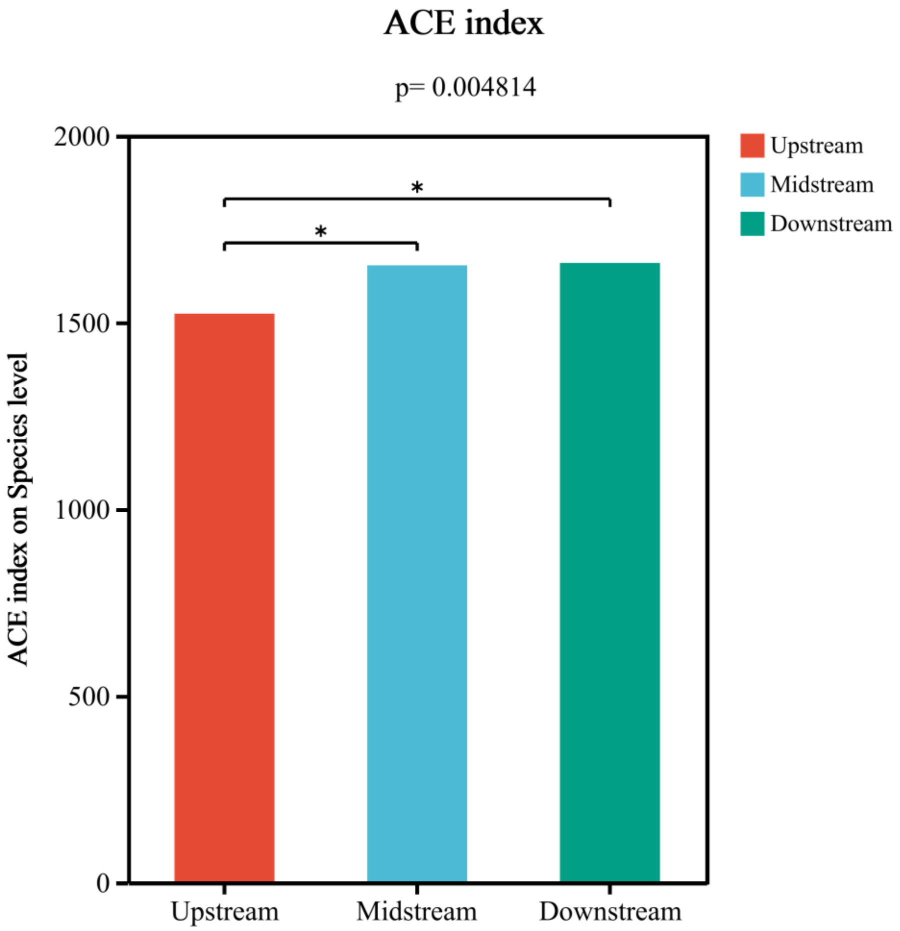

Figure 2b:

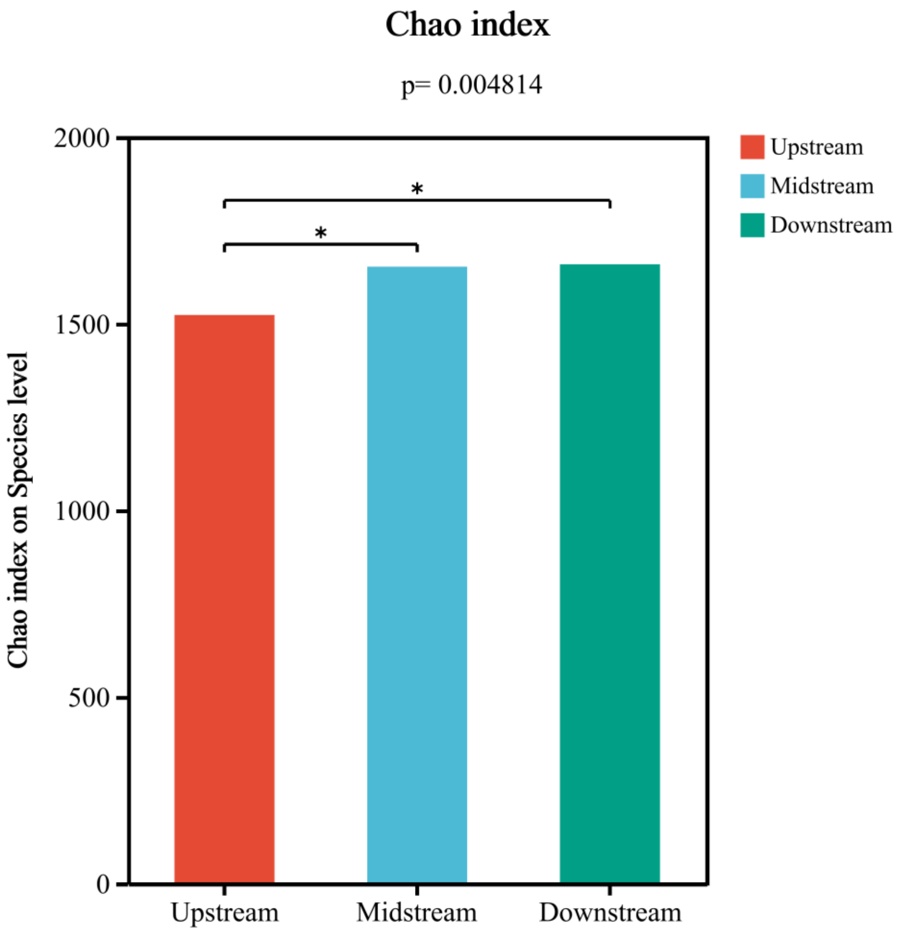
Chao index
p= 0.004814
Chao index on Species level
2000
1500
1000
500
0
*
*
Upstream
Midstream
Downstream
Upstream
Midstream
Downstream

Figure 2c:

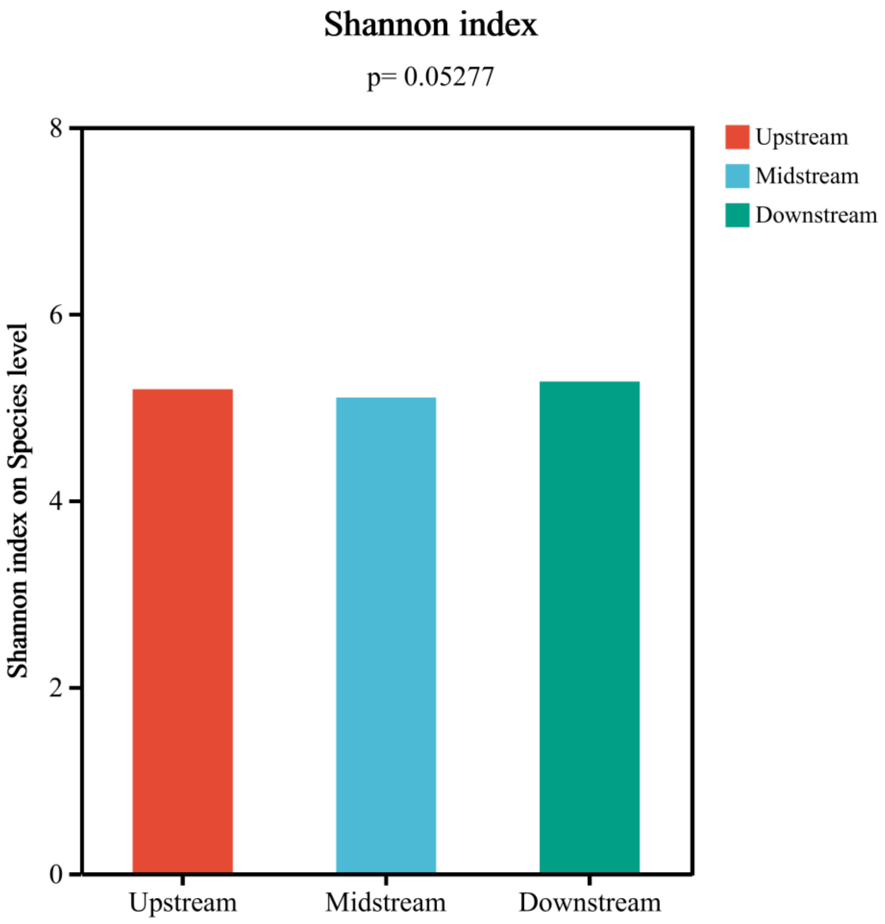

Shannon index
p= 0.05277
Shannon index on Species level
8
6
4
2
0
Upstream
Midstream
Downstream
Upstream
Midstream
Downstream

Figure 2d:

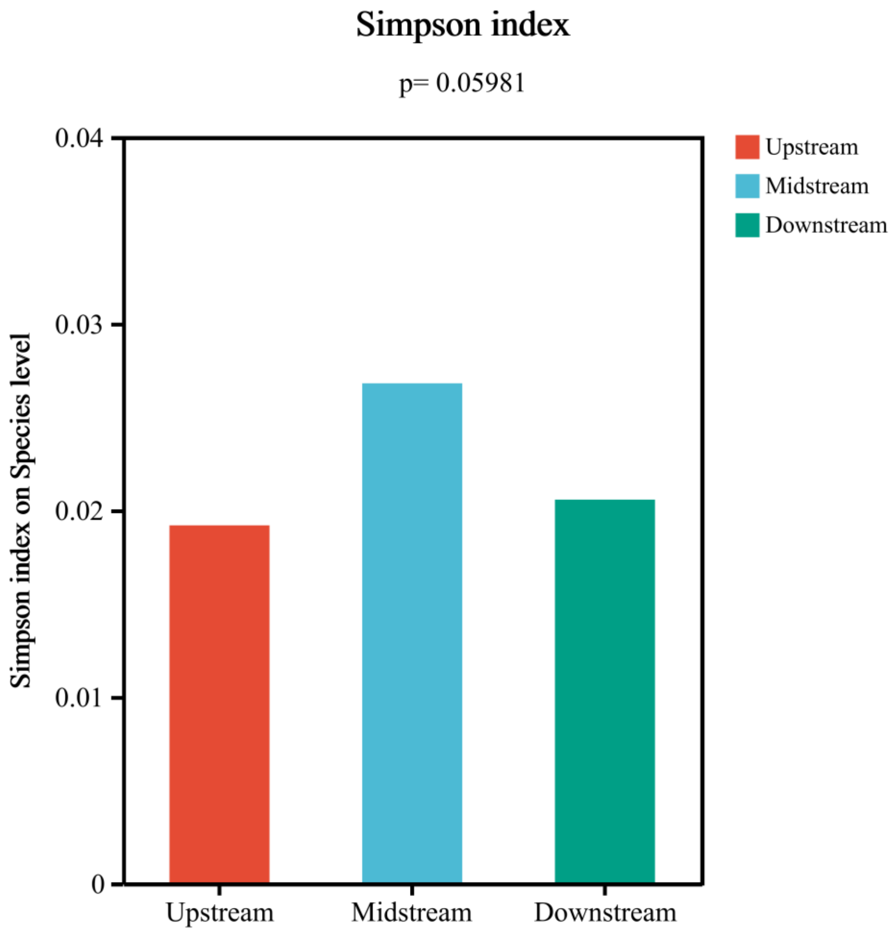
Simpson index
p= 0.05981
0.04
0.03
0.02
0.01
0
Simpson index on Species level
Upstream
Midstream
Downstream
Upstream
Midstream
Downstream

Figure 3a:

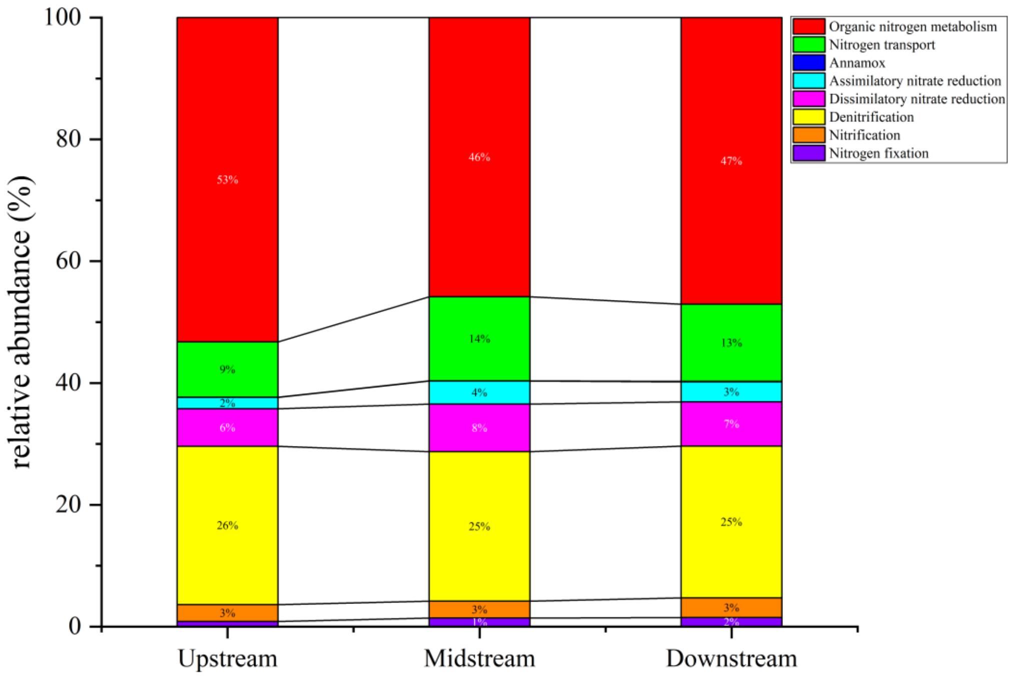

Figure 3b:

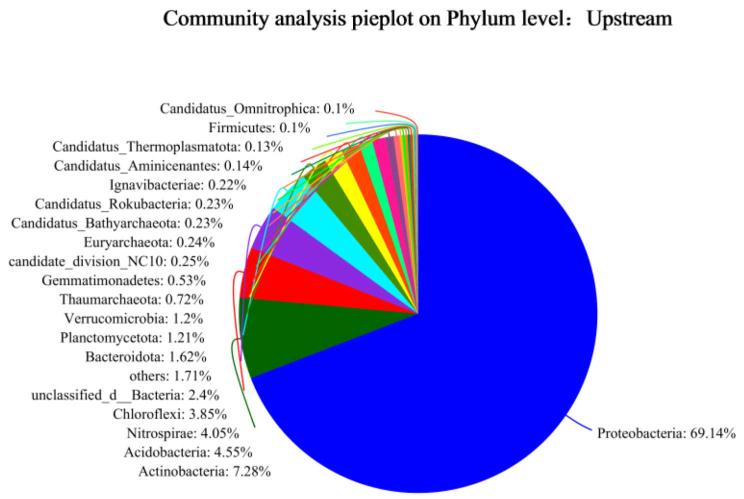

Figure 3c:

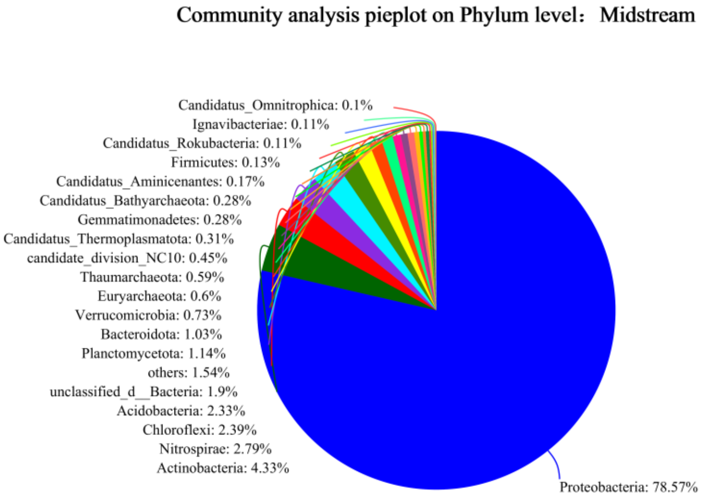

Figure 3d:

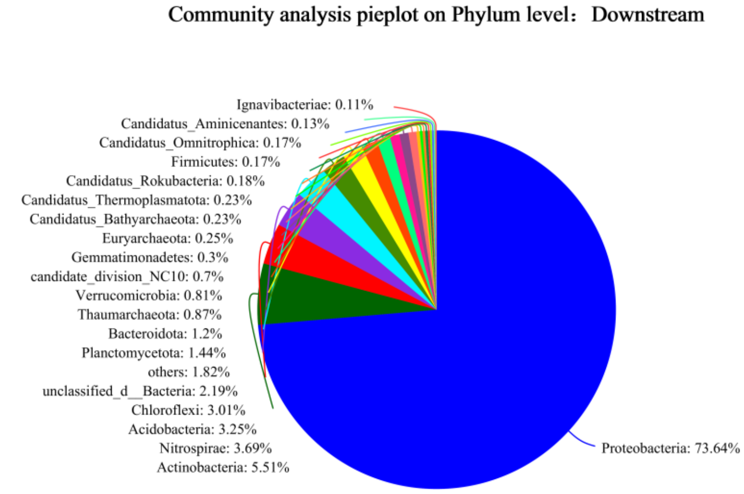

Figure 4a:

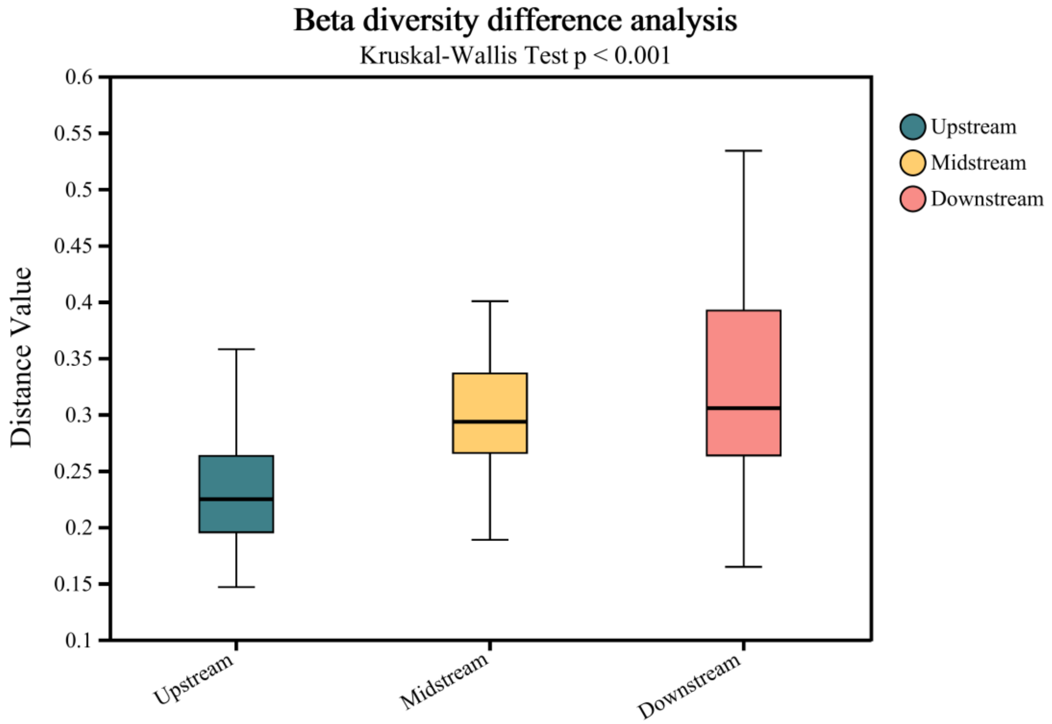

Figure 4b:

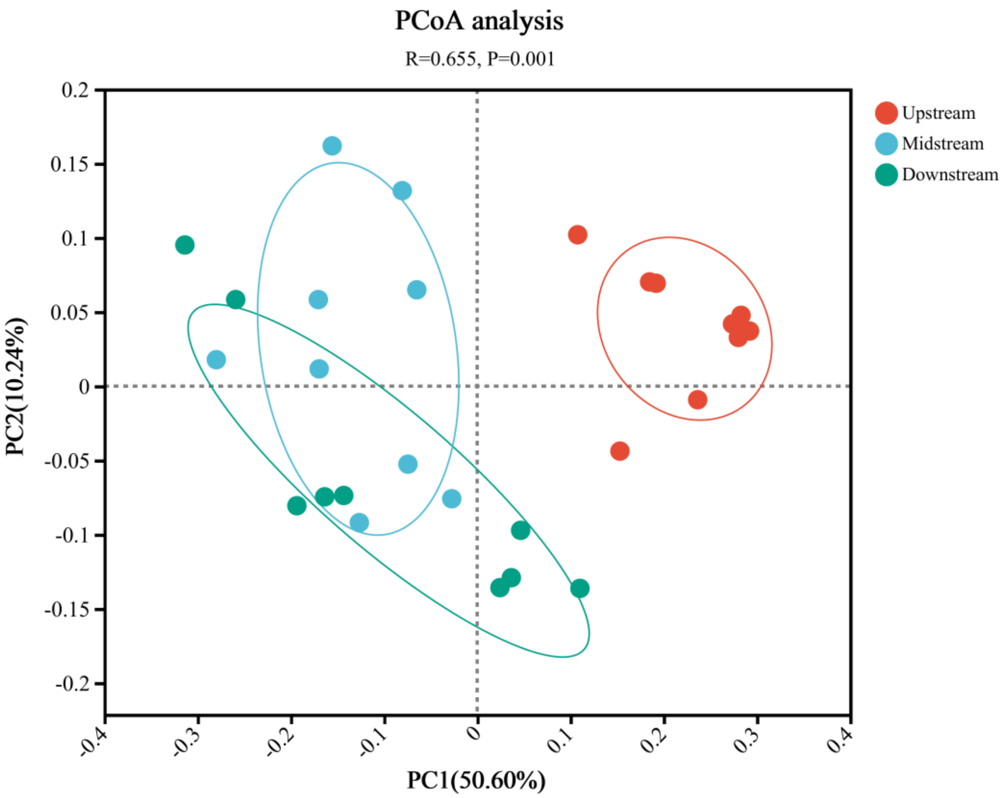

Figure 4c:

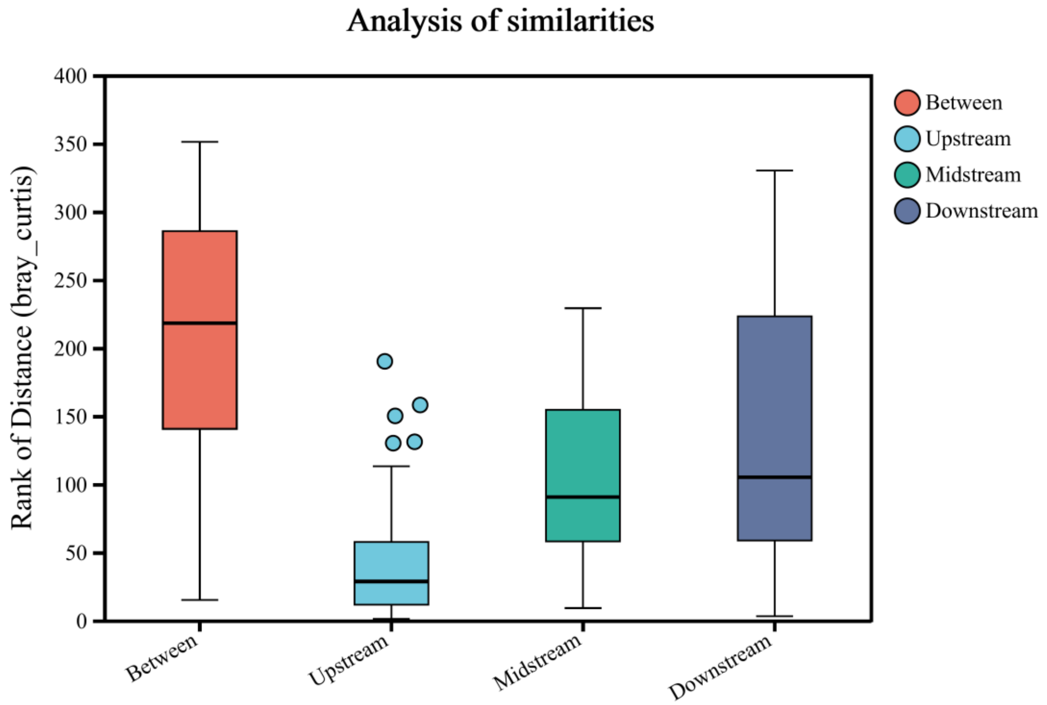

Figure 4d:

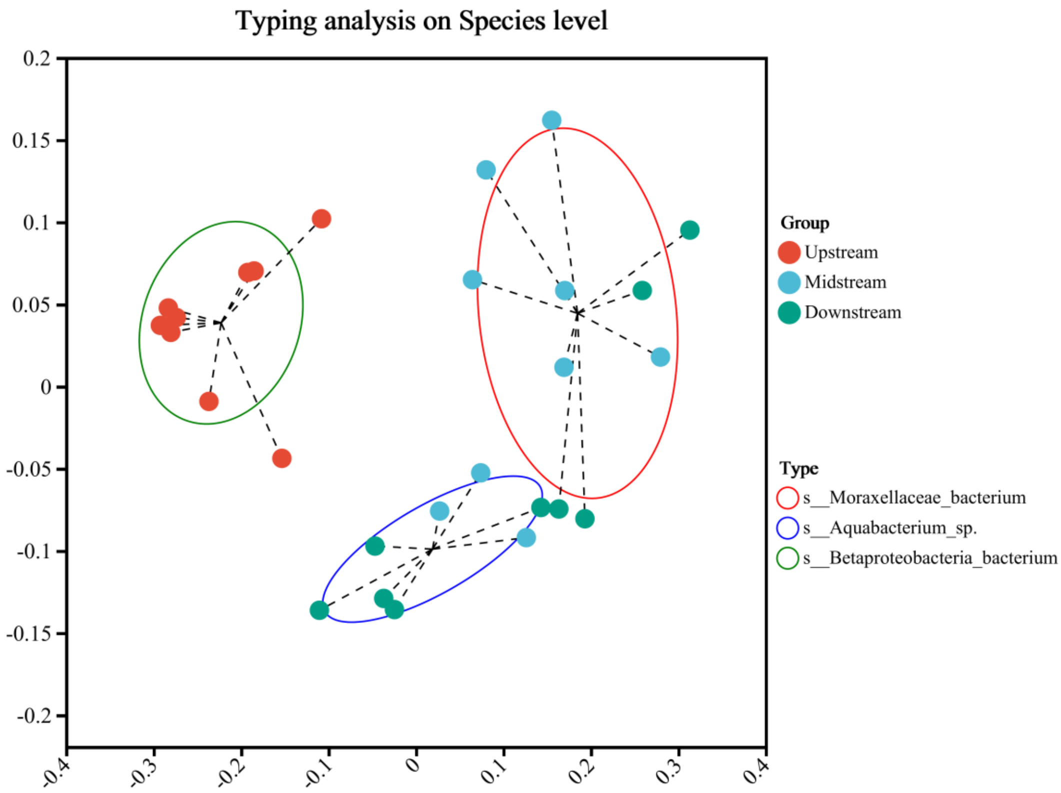

Figure 5a:

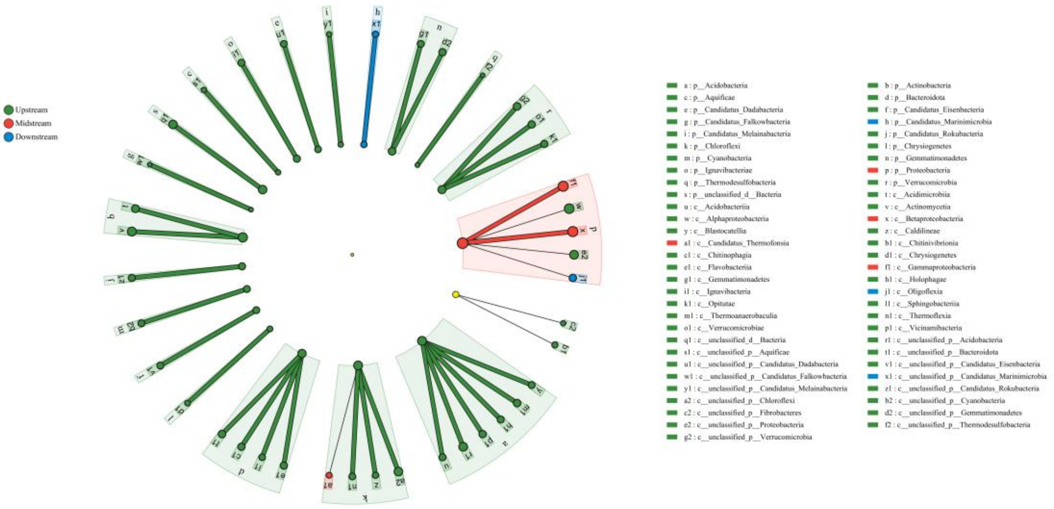

Figure 5b:

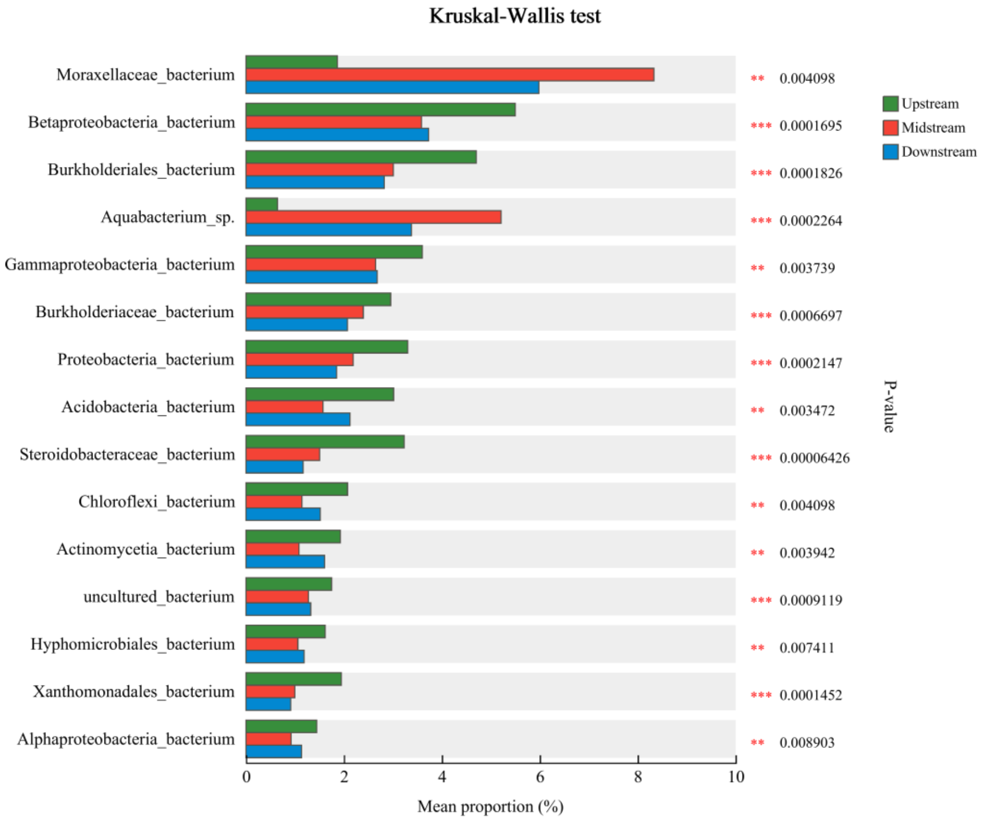

Figure 6a:

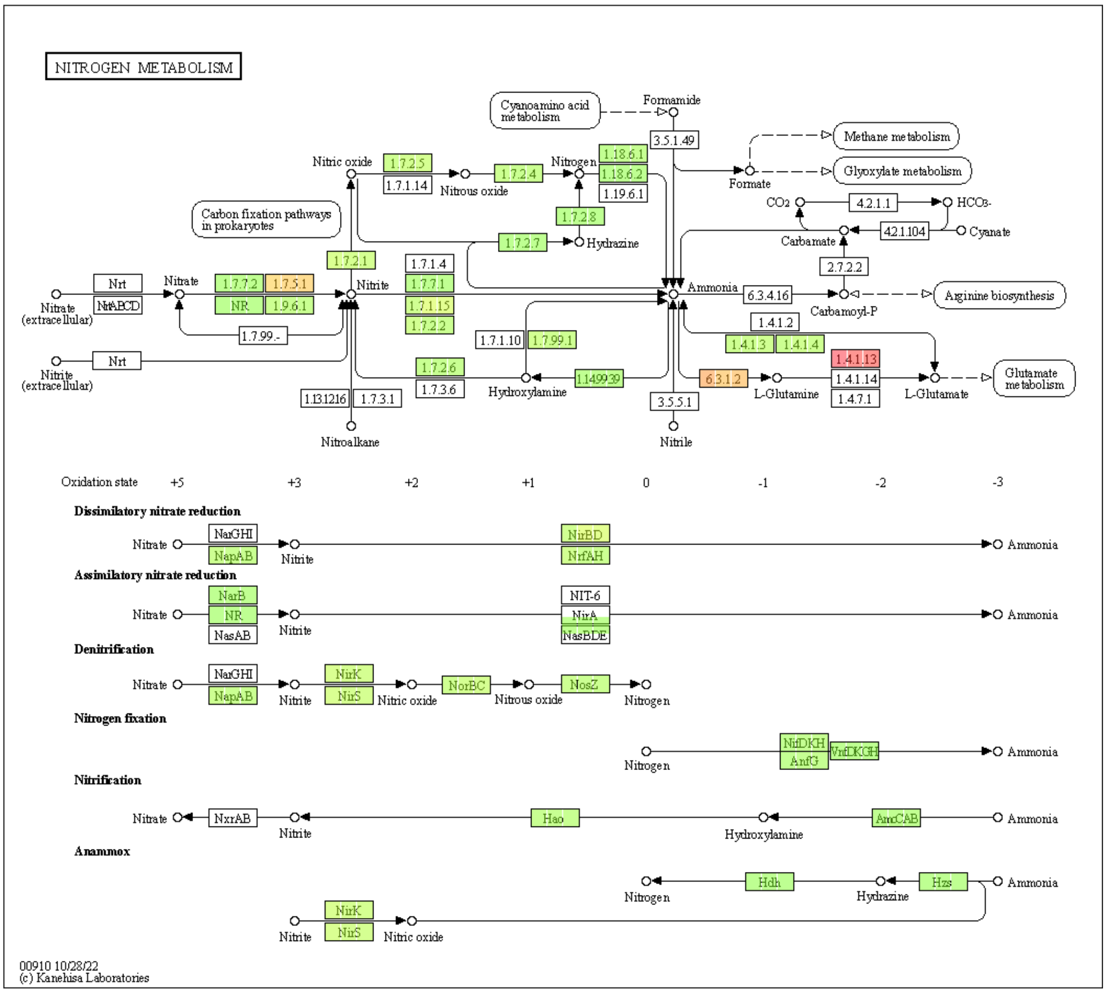
NITROGEN METABOLISM
Cyanoamino acid metabolism
Formamide
3.5.1.49
Methane metabolism
Glyoxylate metabolism
Formate
Nitric oxide
1.7.2.5
1.7.1.14
Nitrous oxide
1.7.2.4
Nitrogen
1.18.6.1
1.18.6.2
1.19.6.1
1.7.2.8
Hydrazine
1.7.2.7
Carbon fixation pathways in prokaryotes
CO2
4.2.1.1
HCO3-
4.2.1.104
Cyanate
Carbamate
2.7.2.2
1.7.2.1
1.7.1.4
Nitrate (extracellular)
Nrt
NrtABCD
Nitrate
1.7.7.2
1.7.5.1
NR
1.9.6.1
Nitrite
1.7.7.1
1.7.1.15
1.7.2.2
Ammonia
6.3.4.16
Carbamoyl-P
Arginine biosynthesis
1.7.99.-
Nitrite (extracellular)
Nrt
1.4.1.2
1.7.1.10
1.7.99.1
1.4.1.3
1.4.1.4
1.4.1.13
1.7.2.6
1.14.99.39
6.3.1.2
1.4.1.14
1.4.7.1
Glutamate metabolism
1.13.12.16
1.7.3.1
1.7.3.6
Hydroxylamine
3.5.5.1
L-Glutamine
L-Glutamate
Nitroalkane
Nitrile
Oxidation state
+5
+3
+2
+1
0
-1
-2
-3
Dissimilatory nitrate reduction
Nitrate
NarGHI
NapAB
Nitrite
NirBD
NrfAH
Ammonia
Assimilatory nitrate reduction
Nitrate
NarB
NR
NasAB
Nitrite
NIT-6
NirA
NasBDE
Ammonia
Denitrification
Nitrate
NarGHI
NapAB
Nitrite
NirK
NirS
Nitric oxide
NorBC
Nitrous oxide
NosZ
Nitrogen
Nitrogen fixation
Nitrogen
NifDKH
AnfG
VnfDKGH
Ammonia
Nitrification
Nitrate
NxrAB
Nitrite
Hao
Hydroxylamine
AmoCAB
Ammonia
Anammox
Nitrogen
Hdh
Hydrazine
Hzs
Ammonia
Nitrite
NirK
NirS
Nitric oxide
00910 10/28/22
(c) Kanehisa Laboratories

Figure 6b:

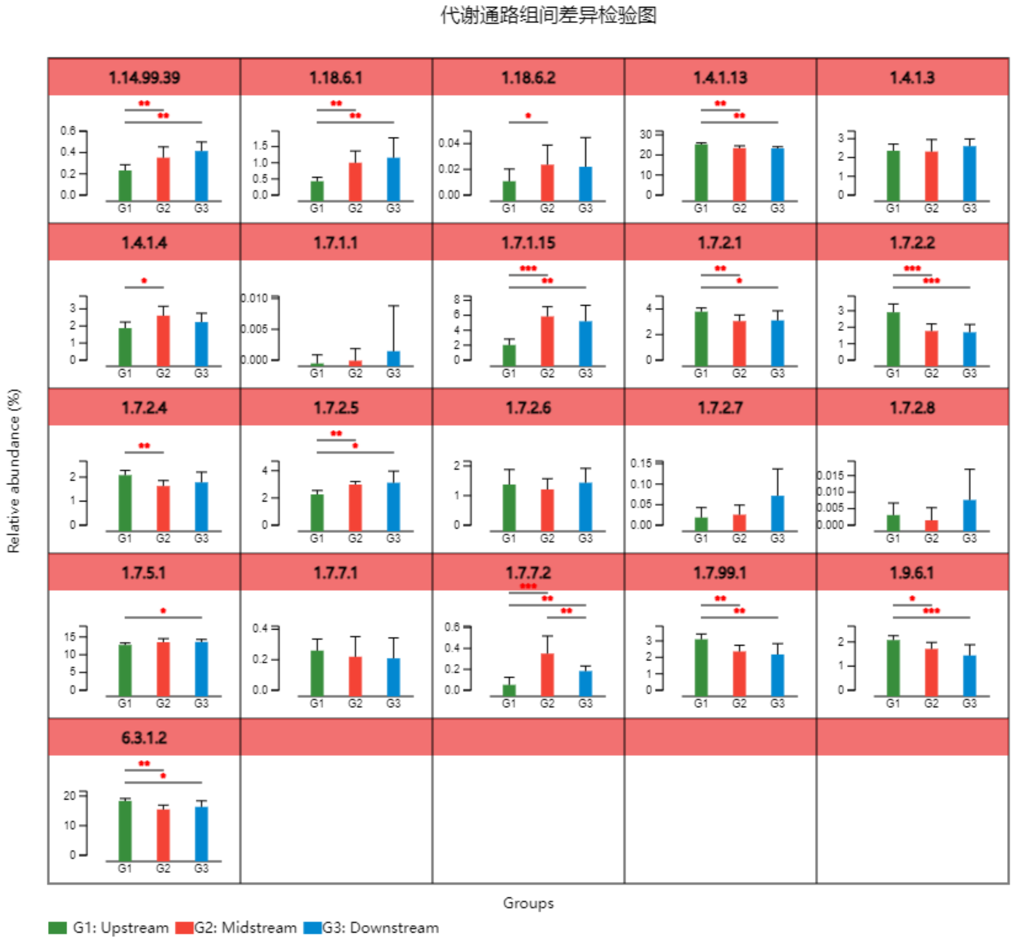
代谢通路组间差异检验图
1.14.99.39
1.18.6.1
1.18.6.2
1.4.1.13
1.4.1.3
1.4.1.4
1.7.1.1
1.7.1.15
1.7.2.1
1.7.2.2
1.7.2.4
1.7.2.5
1.7.2.6
1.7.2.7
1.7.2.8
1.7.5.1
1.7.7.1
1.7.7.2
1.7.99.1
1.9.6.1
6.3.1.2
Relative abundance (%)
Groups
G1: Upstream
G2: Midstream
G3: Downstream

Figure 7a:

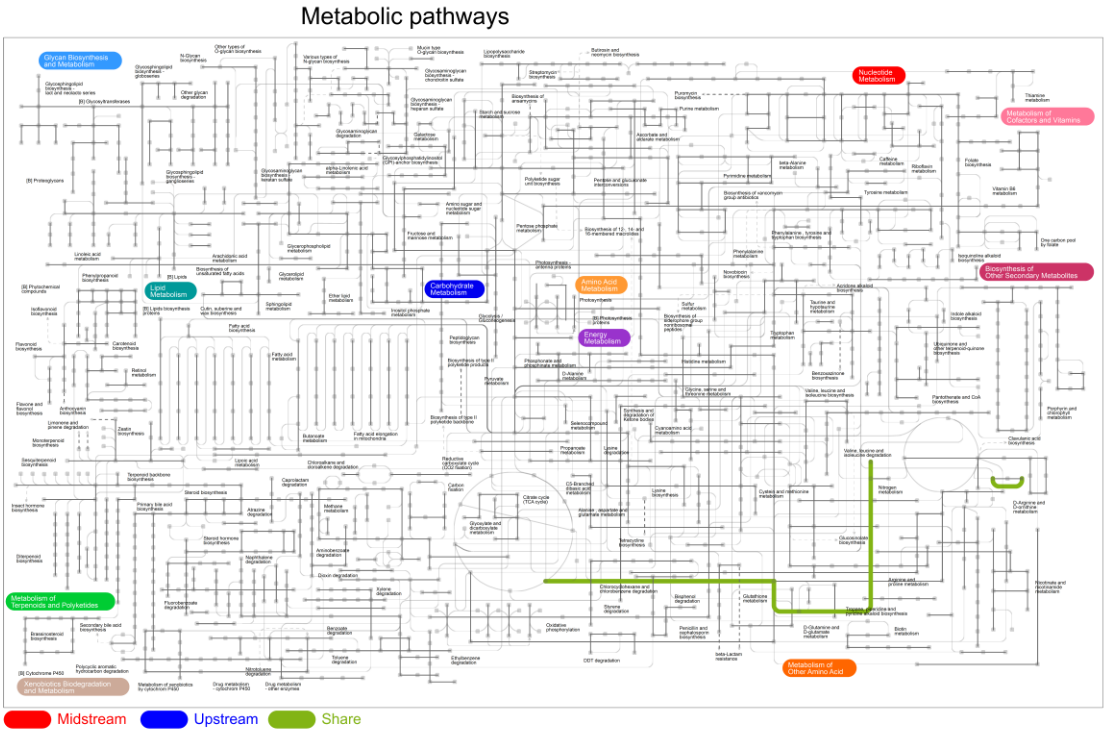

Figure 7b:

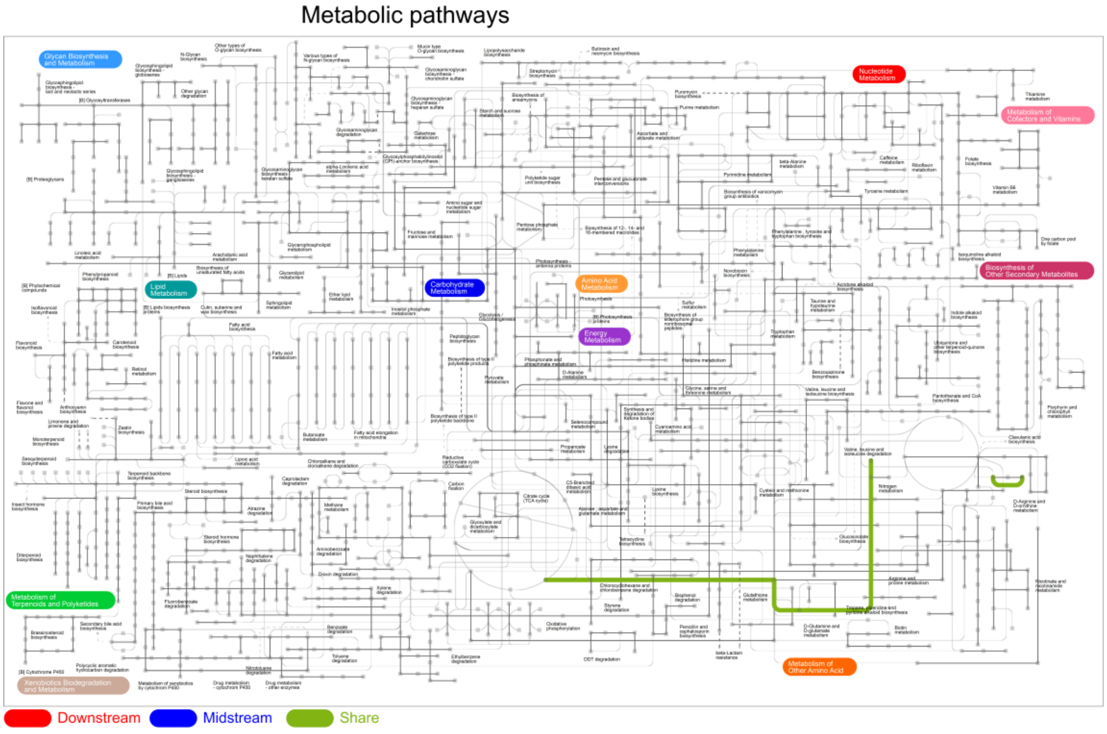

Figure 7c:

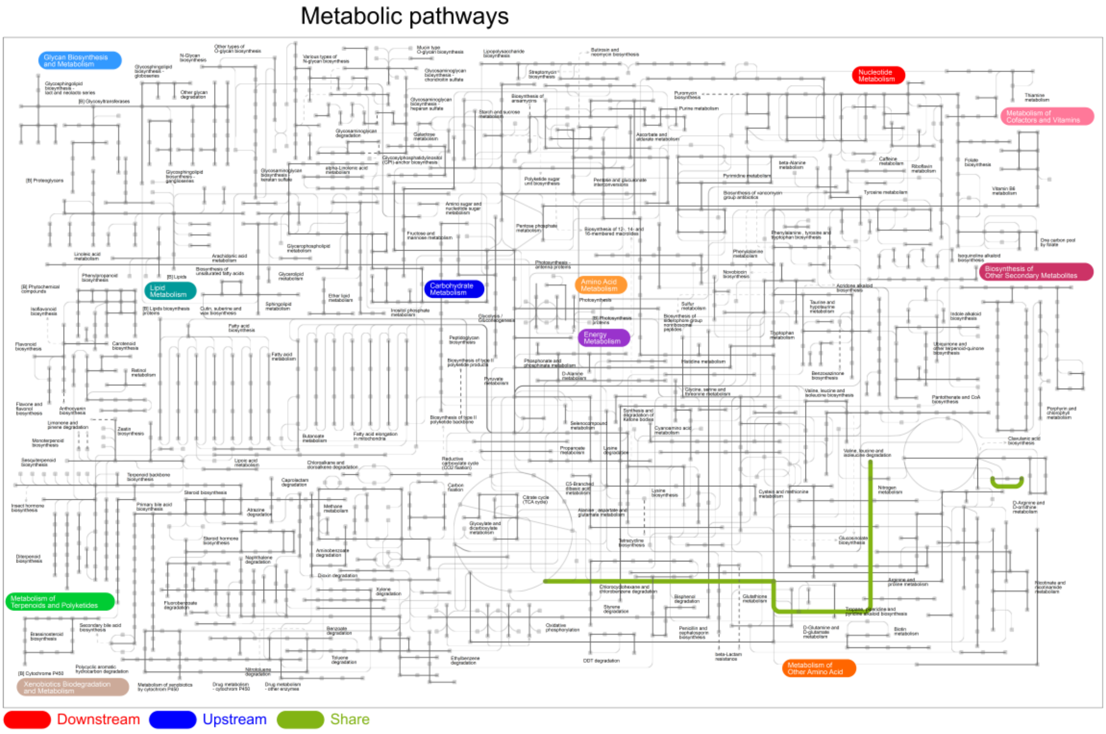

Figure 8a:

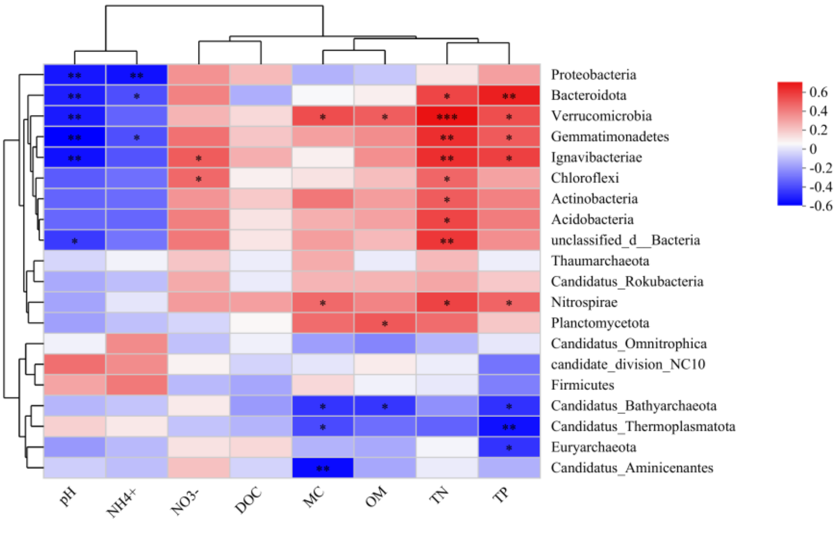

Figure 8b:

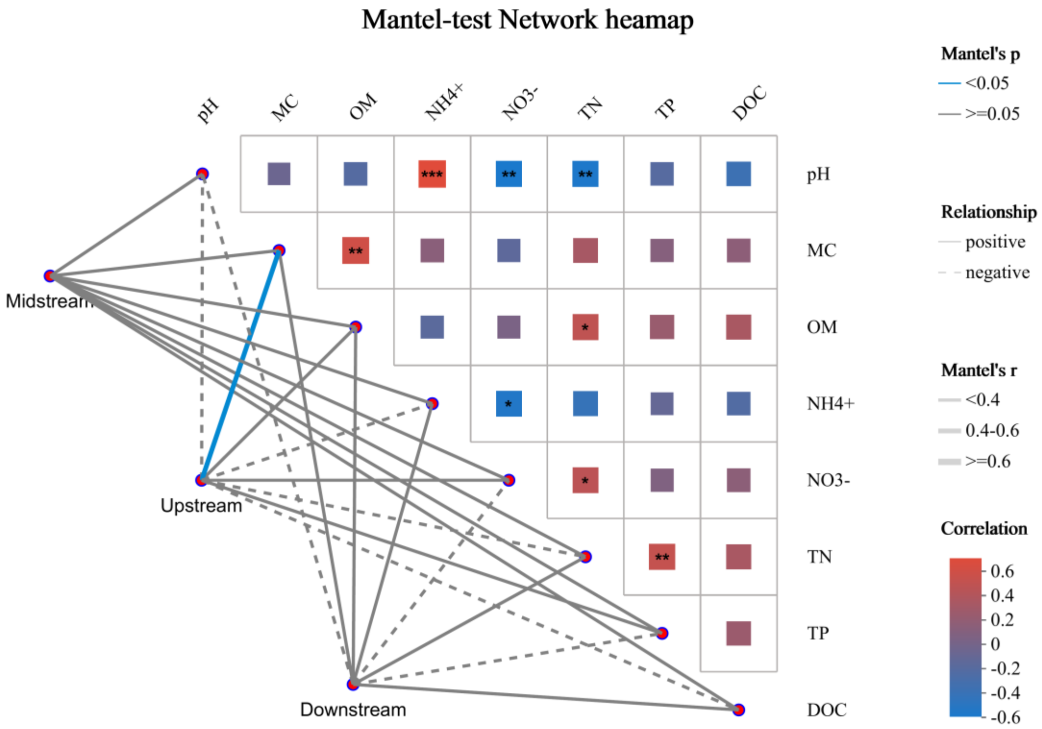

Figure 9a:

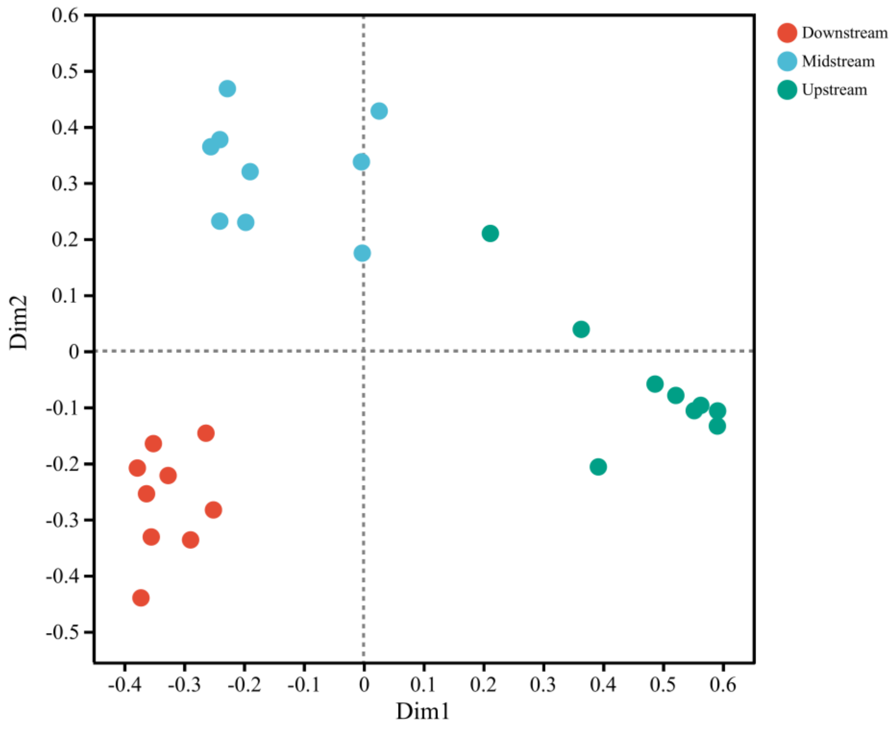

Figure 9b:

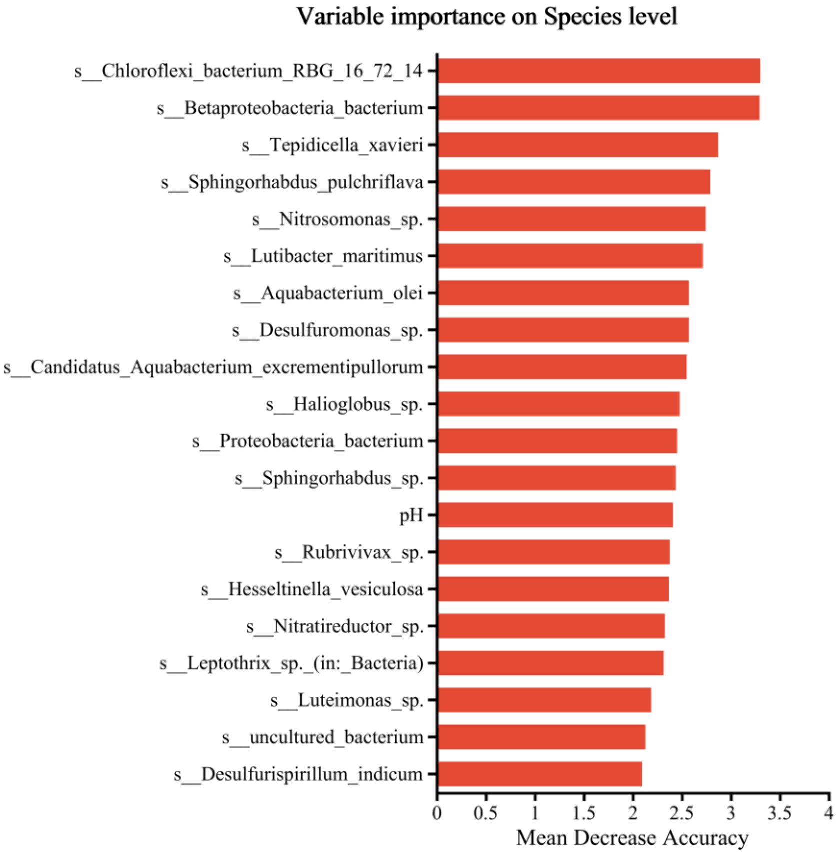

Variable importance on Species level
s__Chloroflexi_bacterium_RBG_16_72_14
s__Betaproteobacteria_bacterium
s__Tepidicella_xavieri
s__Sphingorhabdus_pulchriflava
s__Nitrosomonas_sp.
s__Lutibacter_maritimus
s__Aquabacterium_olei
s__Desulfuromonas_sp.
s__Candidatus_Aquabacterium_excrementipullorum
s__Halioglobus_sp.
s__Proteobacteria_bacterium
s__Sphingorhabdus_sp.
pH
s__Rubrivivax_sp.
s__Hesseltinella_vesiculosa
s__Nitratireductor_sp.
s__Leptothrix_sp._(in:_Bacteria)
s__Luteimonas_sp.
s__uncultured_bacterium
s__Desulfurispirillum_indicum
0
0.5
1
1.5
2
2.5
3
3.5
4
Mean Decrease Accuracy

Figure 10a:

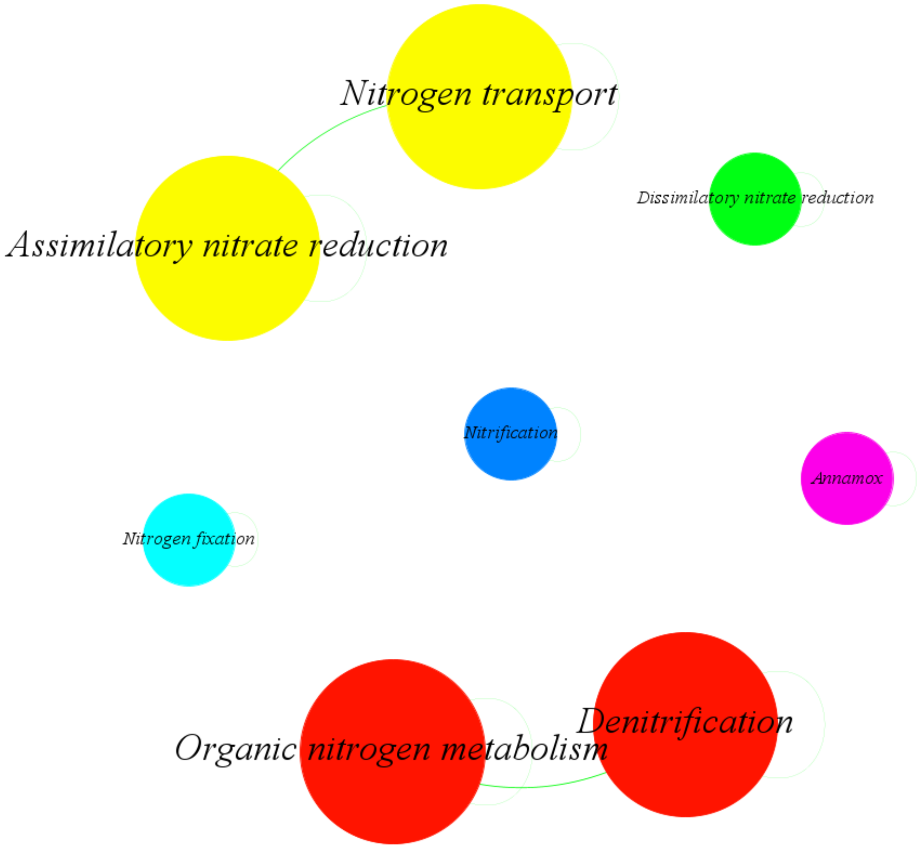
Nitrogen transport
Dissimilatory nitrate reduction
Assimilatory nitrate reduction
Nitrification
Annamox
Nitrogen fixation
Organic nitrogen metabolism
Denitrification

Figure 10b:

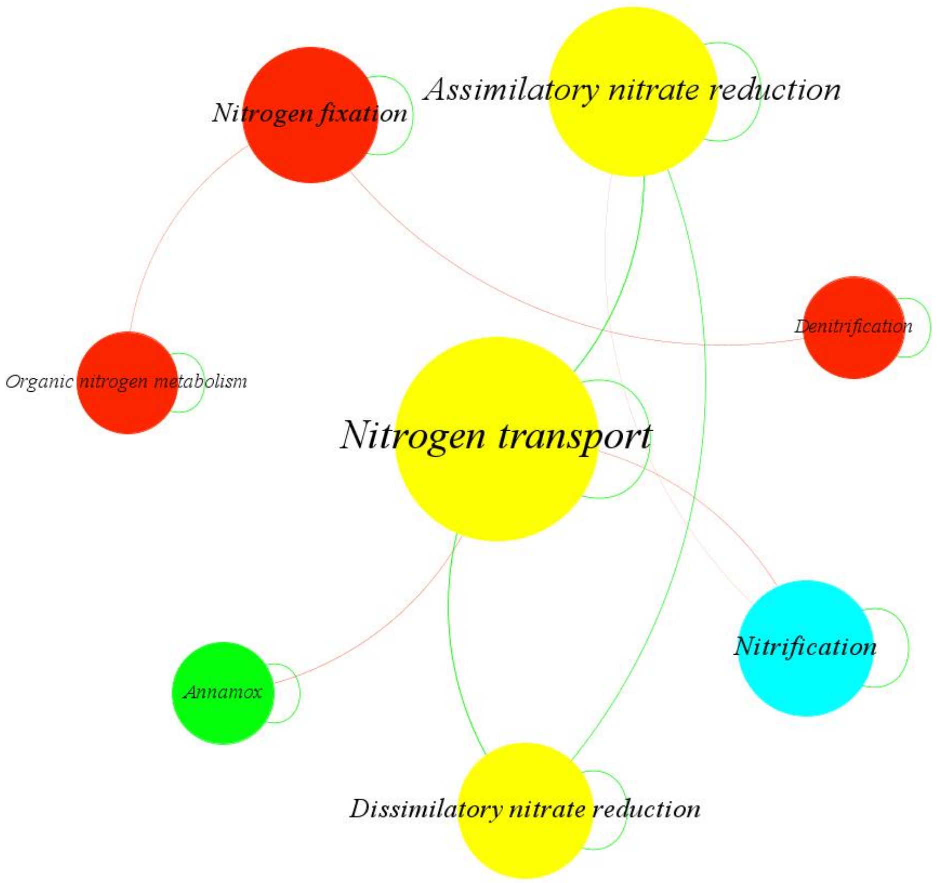
Nitrogen fixation
Assimilatory nitrate reduction
Denitrification
Organic nitrogen metabolism
Nitrogen transport
Nitrification
Annamox
Dissimilatory nitrate reduction

Figure 10c:

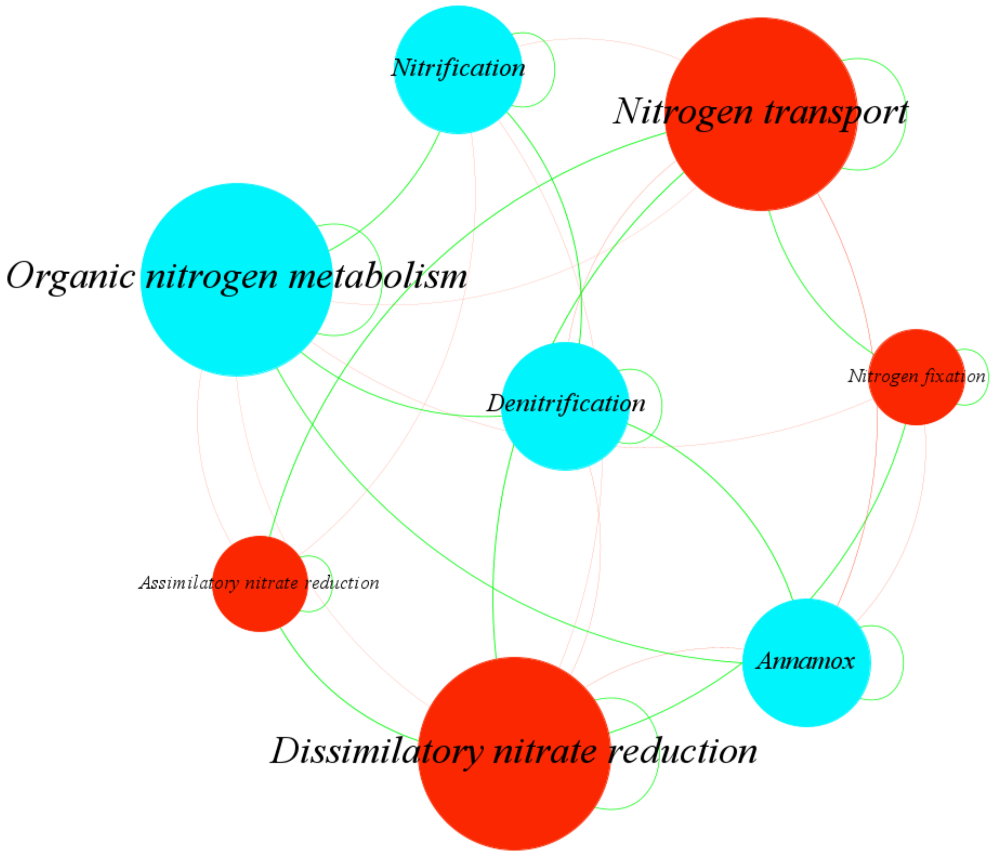
Nitrification
Nitrogen transport
Organic nitrogen metabolism
Nitrogen fixation
Denitrification
Assimilatory nitrate reduction
Annamox
Dissimilatory nitrate reduction

Figure 10d:

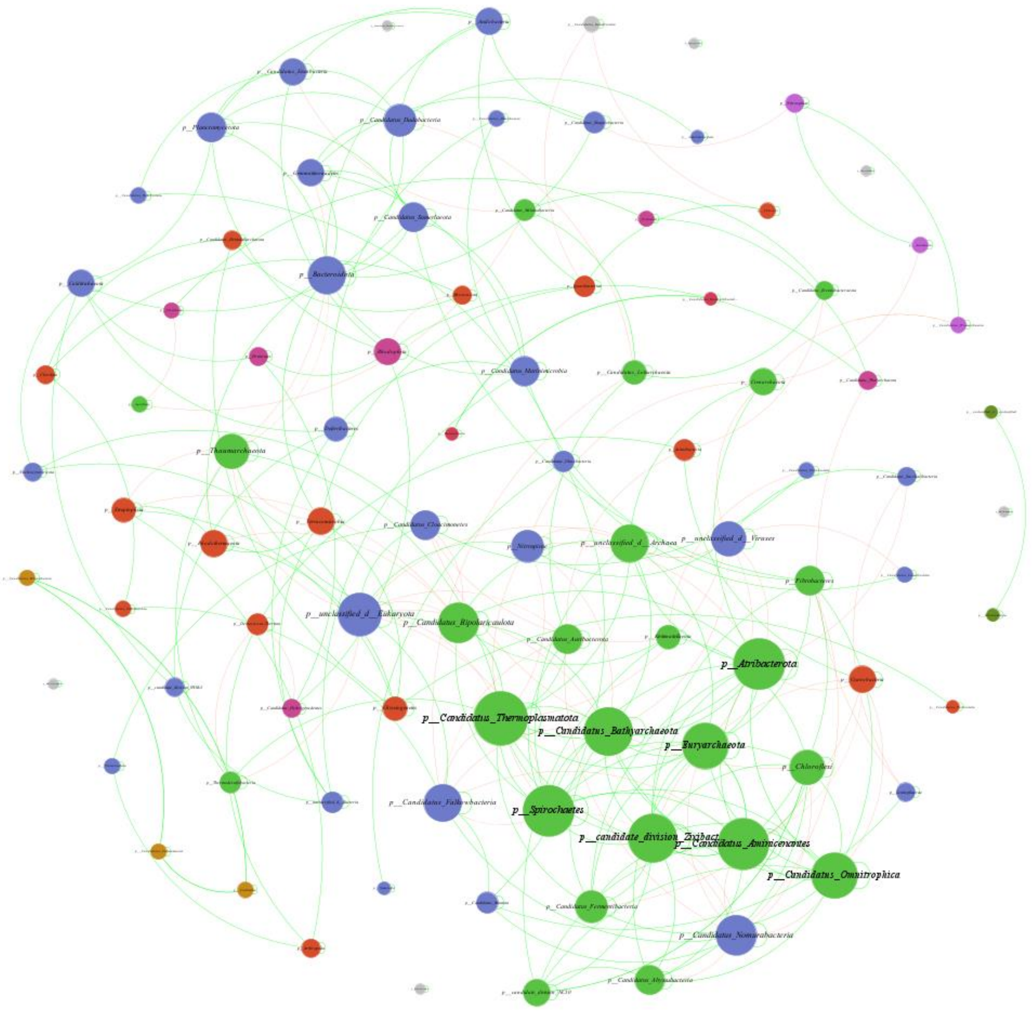
p__Atribacterota
p__Candidatus_Thermoplasmatota
p__Candidatus_Bathyarchaeota
p__Euryarchaeota
p__Spirochaetes
p__Candidatus_Aminicenantes
p__Candidatus_Omnitrophica
p__Candidatus_Nomurabacteria
p__unclassified_d__Eukaryota
p__unclassified_d__Archaea
p__unclassified_d__Viruses
p__Chloroflexi
p__Thaumarchaeota
p__Bacteroidota

Figure 10e:

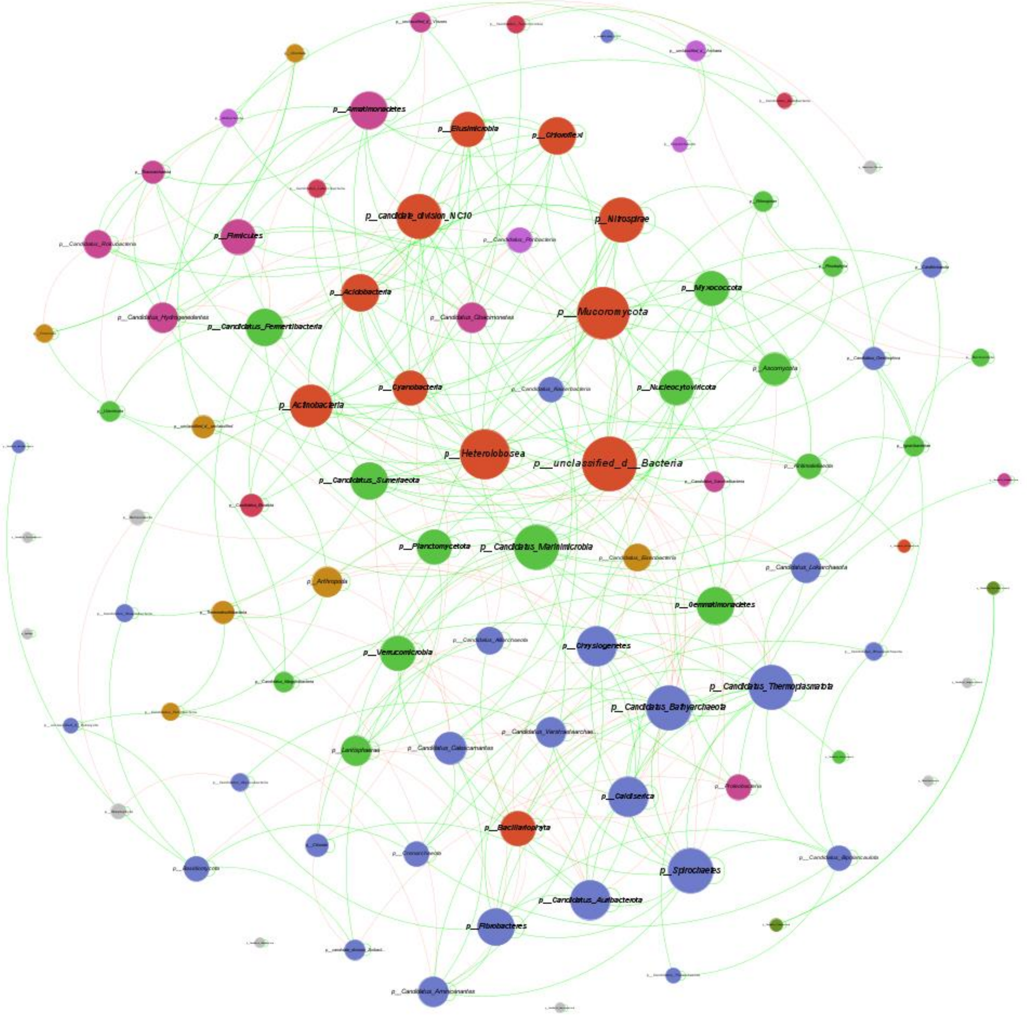

p__Armatimonadetes
p__Elusimicrobia
p__Chloroflexi
p__candidate_division_NC10
p__Nitrospirae
p__Firmicutes
p__Acidobacteria
p__Mucoromycota
p__Myxococcota
p__Candidatus_Fermentibacteria
p__Cyanobacteria
p__Nucleocytoviricota
p__Actinobacteria
p__Heterolobosea
p__unclassified_d__Bacteria
p__Candidatus_Sumerlaeota
p__Planctomycetota
p__Candidatus_Marinimicrobia
p__Arthropoda
p__Gemmatimonadetes
p__Verrucomicrobia
p__Chrysiogenetes
p__Candidatus_Thermoplasmatota
p__Candidatus_Bathyarchaeota
p__Calditrica
p__Bacillariophyta
p__Spirochaetes
p__Candidatus_Auribacterota
p__Fibrobacteres

Figure 10f:

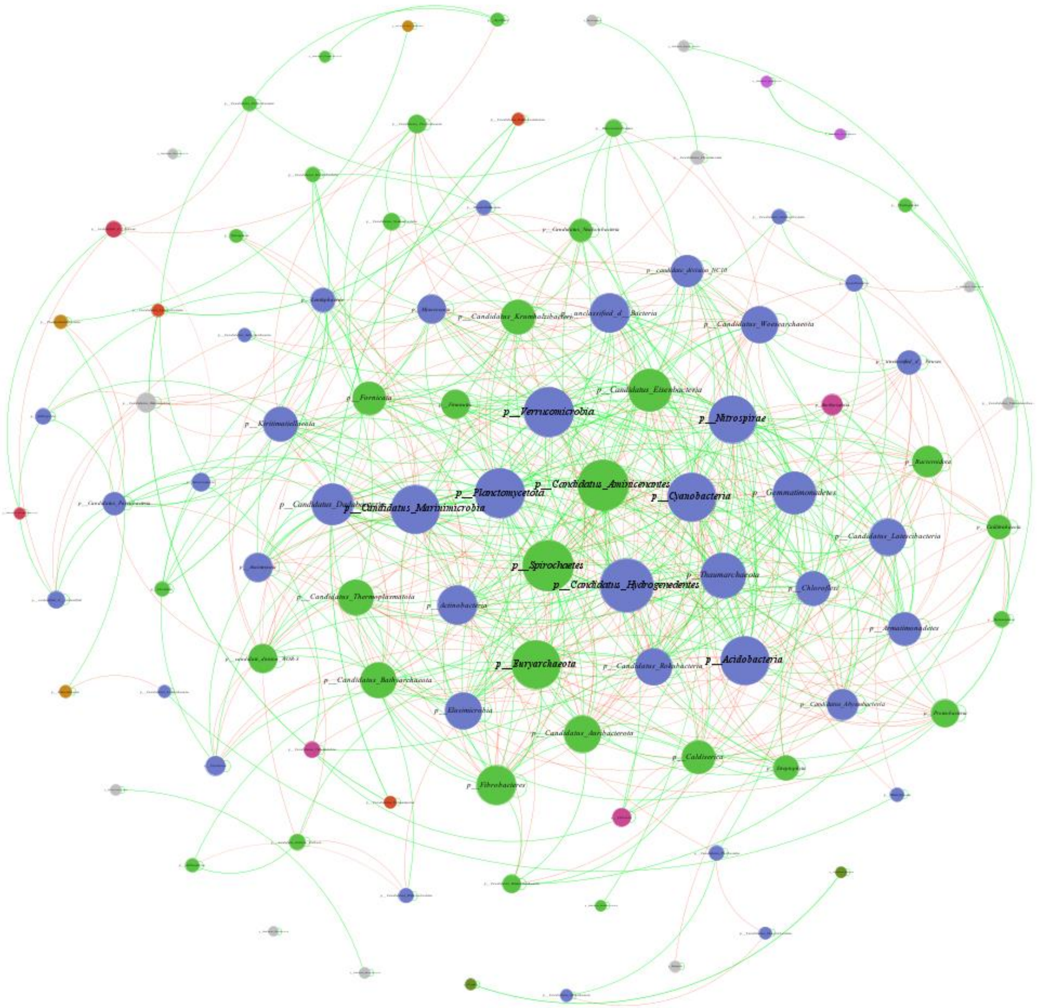
p__Verrucomicrobia
p__Nitrospirae
p__Candidatus_Aminicenantes
p__Planctomycetota
p__Candidatus_Marinimicrobia
p__Cyanobacteria
p__Gemmatimonadetes
p__Spirochaetes
p__Candidatus_Hydrogenedentes
p__Thaumarchaeota
p__Chloroflexi
p__Euryarchaeota
p__Acidobacteria
p__Candidatus_Rokubacteria
p__Candidatus_Bathyarchaeota
p__Candidatus_Thermoplasmatota
p__Actinobacteria
p__Elusimicrobia
p__Candidatus_Aerophobota
p__Fibrobacteres
p__Caldiserica
p__Candidatus_Eisenbacteria
p__Fornicata
p__Kiritimatiellaeota
p__unclassified_d__Bacteria
p__candidate_division_NC10
p__Candidatus_Woesearchaeota
p__Candidatus_Latescibacteria
p__Armatimonadetes
p__Candidatus_Abyssubacteria
p__Bacteroidota
