# Supplementary material for: Characterizing nitrogen cycling microorganisms and genes in sediments of the Three Gorges Reservoir
Source: PLoS One. 2025 Jun 10;20(6):e0324051. doi: 10.1371/journal.pone.0324051 (PMC12151472; doi:10.1371/journal.pone.0324051)
Supplement: Attachment — Submitted filename: Response to Reviewers.docx [file pone.0324051.s004.docx]

Response to Reviewers

Journal requirements: When submitting your revision, we need you to address these additional requirements.

1. Please ensure that your manuscript meets PLOS ONE's style requirements, including those for file naming. The PLOS ONE style templates can be found at https://journals.plos.org/plosone/s/file?id=wjVg/PLOSOne_formatting_sample_main_body.pdf and <https://journals.plos.org/plosone/s/file?id=ba62/PLOSOne_formatting_sample_title_authors_affiliations.pdf>
2. In your Methods section, please provide additional information regarding the permits you obtained for the work. Please ensure you have included the full name of the authority that approved the field site access and, if no permits were required, a brief statement explaining why.
3. Thank you for stating the following financial disclosure: “The authors gratefully acknowledge the financial support provided by the Key Project of the National Natural Science Foundation of China (52130903).” Please state what role the funders took in the study.  If the funders had no role, please state: "The funders had no role in study design, data collection and analysis, decision to publish, or preparation of the manuscript." If this statement is not correct you must amend it as needed. Please include this amended Role of Funder statement in your cover letter; we will change the online submission form on your behalf.
4. When completing the data availability statement of the submission form, you indicated that you will make your data available on acceptance. We strongly recommend all authors decide on a data sharing plan before acceptance, as the process can be lengthy and hold up publication timelines. Please note that, though access restrictions are acceptable now, your entire data will need to be made freely accessible if your manuscript is accepted for publication. This policy applies to all data except where public deposition would breach compliance with the protocol approved by your research ethics board. If you are unable to adhere to our open data policy, please kindly revise your statement to explain your reasoning and we will seek the editor's input on an exemption. Please be assured that, once you have provided your new statement, the assessment of your exemption will not hold up the peer review process.
5. PLOS requires an ORCID iD for the corresponding author in Editorial Manager on papers submitted after December 6th, 2016. Please ensure that you have an ORCID iD and that it is validated in Editorial Manager. To do this, go to ‘Update my Information’ (in the upper left-hand corner of the main menu), and click on the Fetch/Validate link next to the ORCID field. This will take you to the ORCID site and allow you to create a new iD or authenticate a pre-existing iD in Editorial Manager.
6. We note that Figure 1 in your submission contain [map/satellite] images which may be copyrighted. All PLOS content is published under the Creative Commons Attribution License (CC BY 4.0), which means that the manuscript, images, and Supporting Information files will be freely available online, and any third party is permitted to access, download, copy, distribute, and use these materials in any way, even commercially, with proper attribution. For these reasons, we cannot publish previously copyrighted maps or satellite images created using proprietary data, such as Google software (Google Maps, Street View, and Earth). For more information, see our copyright guidelines: http://journals.plos.org/plosone/s/licenses-and-copyright. We require you to either (1) present written permission from the copyright holder to publish these figures specifically under the CC BY 4.0 license, or (2) remove the figures from your submission: a. You may seek permission from the original copyright holder of Figure 1 to publish the content specifically under the CC BY 4.0 license.  We recommend that you contact the original copyright holder with the Content Permission Form (http://journals.plos.org/plosone/s/file?id=7c09/content-permission-form.pdf) and the following text: “I request permission for the open-access journal PLOS ONE to publish XXX under the Creative Commons Attribution License (CCAL) CC BY 4.0 (http://creativecommons.org/licenses/by/4.0/). Please be aware that this license allows unrestricted use and distribution, even commercially, by third parties. Please reply and provide explicit written permission to publish XXX under a CC BY license and complete the attached form.” Please upload the completed Content Permission Form or other proof of granted permissions as an ""Other"" file with your submission. In the figure caption of the copyrighted figure, please include the following text: “Reprinted from [ref] under a CC BY license, with permission from [name of publisher], original copyright [original copyright year].” b. If you are unable to obtain permission from the original copyright holder to publish these figures under the CC BY 4.0 license or if the copyright holder’s requirements are incompatible with the CC BY 4.0 license, please either i) remove the figure or ii) supply a replacement figure that complies with the CC BY 4.0 license. Please check copyright information on all replacement figures and update the figure caption with source information. If applicable, please specify in the figure caption text when a figure is similar but not identical to the original image and is therefore for illustrative purposes only. The following resources for replacing copyrighted map figures may be helpful: USGS National Map Viewer (public domain): http://viewer.nationalmap.gov/viewer/ The Gateway to Astronaut Photography of Earth (public domain): http://eol.jsc.nasa.gov/sseop/clickmap/ Maps at the CIA (public domain): https://www.cia.gov/library/publications/the-world-factbook/index.html and https://www.cia.gov/library/publications/cia-maps-publications/index.html NASA Earth Observatory (public domain): http://earthobservatory.nasa.gov/ Landsat: http://landsat.visibleearth.nasa.gov/ USGS EROS (Earth Resources Observatory and Science (EROS) Center) (public domain): http://eros.usgs.gov/# Natural Earth (public domain): <http://www.naturalearthdata.com/>
7. Please remove your figures from within your manuscript file, leaving only the individual TIFF/EPS image files, uploaded separately. These will be automatically included in the reviewers’ PDF.

**Reply:**

1. I have found the style template for the journal on two websites and modified the manuscript according to the template style to ensure it meets the journal's style requirements.
2. No permits were required for this study as the research was conducted in public areas and within regions that do not necessitate specific authorization for the type of data collection and analysis we performed. Our work adhered to all ethical guidelines and did not involve any activities that would require special permissions or approvals.
3. The funder had no role in study design, data collection and analysis, decision to publish, or preparation of the manuscript. And I have already written the amended role of funder statement in my cover letter.
4. Before acceptance, I will adopt the data sharing plan and comply with the open data policy.
5. I possess an ORCID iD and have successfully completed the validation process within Editorial Manager.
6. I have deleted Figure 1.
7. I have removed all the figures from my manuscript and uploaded them separately as attachments.

Reviewer #1: The manuscript ‘Characterizing Nitrogen-Cycling Microorganisms and Genes in Sediments of the Three Gorges Reservoir’ focuses an important topic of investigating microbial-driven nitrogen cycle processes in the sediments of the Three Gorges Reservoir and assess the overall state of nitrogen cycling. The current manuscript comes in the domain of the journal and I appreciate the authors for their research work. However, due to various issues across different sections of manuscript, it needs revision. Overall, sentence structure needs to be improved. Some of general comments/questions are listed below while more specific questions and comments are inserted in the attached PDF file.

In abstract elaborate briefly, what authors want to say when write ‘46 types of nitrogen conversion functional genes’, nitrogen conversion not accurate there. Further. it’s not ‘refine’ rather this study aims to investigate or report. Please rephrase. The current structure of the introduction is not well organized. Additionally, the last part of the introduction needs to be revised, considering the main theme/objectives and findings of the study. Materials and Methods are very descriptive, it’s more like a thesis document especially in methods section, there are a large number of sub-headings in methods. And the representation of data also needs to be extensively improved. Further, the figures could be merged, or only key data may be included instead of having huge numbers which could easily be merged. The results are explained while the Discussion section could be improved, for example in addition to discussing the results in line with previous reports, key findings need to be discussed and highlighted. The section 4 overall, and specifically with focus on ‘ammonia oxidation requires specific environmental conditions’ is also like a thesis document, much detail and redundant rather than just discussing the key findings. Another very crucial aspect of the work i.e. how the ‘variations in sediment particle size’ influences the N cycling is described but lacks in depth analyses, for example, the relative abundance of these functional guilds across various environments could also be compared to enlighten the current results. Again, like other sections of the manuscript, Discussion, and Conclusions sections also needs to be comprehensive (instead of detailed paras and redundancy at places), the focus could only be on the key findings of the work.

**Reply:**

I have already made modifications to all the problematic areas listed in the PDF file according to the comments.

I have rewritten the abstract section, reorganizing the language. The sentence "46 types of nitrogen conversion functional genes" has been removed, and the structure of the sentences has been reorganized to ensure accurate expression.

I have revised the last part of the introduction, incorporating the research theme/objectives and the investigation results.

I have simplified the descriptions in the "Materials and Methods" section and consolidated some overly detailed sub-headings to reduce the complexity of the methods part and ensure that each section is directly relevant and contributes to the research findings.

I have eliminated certain analyses that bear relatively low relevance to the overall conclusion and opted to present solely the key data. By doing so, I aim to ensure the effective conveyance of the information's essence while circumventing excessive and redundant data. I have endeavored to render the data presentation more concise and influential, thus conforming to the standards expected of high-quality research papers.

While reviewing the manuscript, I noticed in the section “3.5 Correlative analysis of environmental factors” that some data for the environmental factor had been incorrectly entered during the initial analysis and graphing. I have since input the correct data, conducted a new analysis and redrafted the figures, and included the updated results in the manuscript.

Similarly, due to some incorrect inputs of environmental factors initially, there were also issues with the analysis in the section "3.6 Random forest analysis". Therefore, after inputting the correct data, I conducted a new analysis and redrafted the figures, and have included the updated results in the manuscript.

I have made improvements to the discussion section, focusing on and emphasizing the key findings. I have eliminated redundant parts and conducted an in-depth analysis of how “variations in sediment particle size” influences the nitrogen cycle. The discussion and conclusion sections have been thoroughly revised to highlight the critical findings of this study.

Reviewer #2: The research work described the Characterization of Nitrogen-Cycling Microorganisms and Genes in Sediments. The article reports interesting results, however write up of the article could be a lot better than this draft. Language of introduction could be improved. There are so many sub-sections in the methodology section which do not follow the article style. A huge number of figures are also not desired. The quality of Figure 2a is not readable. Nitrogen is a very crucial element across sediments, discussion section needs to be extensively improved considering the nitrogen importance across sediments.

**Reply:**

I have improved the language in the introduction section. The methods section has been revised by removing unnecessary subsections to streamline the presentation and improve clarity. A significant amount of analysis and figures with weak relevance to this study have been removed. I have enhanced the quality of the figures and included all high-definition figures in the appendix. The discussion section has been thoroughly modified.
